# Tuning Crystal Field and Spin Dynamics in Gd(III) Complexes via Coordination Geometry

**DOI:** 10.1021/acs.inorgchem.6c03603

**Published:** 2026-10-13

**Authors:** Corina Rodríguez-Esteban, Daiana Cabrosi, Beñat Artetxe, Luis Lezama, Mathieu Gonidec, Itziar Oyarzabal

**Affiliations:** † Basque Center for Materials, Applications and Nanostructures, 518636 BCMaterials, 48940 Leioa, Spain; ‡ CNRS, Bordeaux INP, ICMCB, UMR 5026, Université Bordeaux, 33600 Pessac, France; § Departamento de Química Aplicada y Materiales Poliméricos, Facultad de Química, Universidad del País Vasco EHU, 20018 Donostia-San Sebastián, Spain; ∥ Departamento de Química Orgánica e Inorgánica, Facultad de Ciencia y Tecnología, Universidad del País Vasco EHU, 48080 Bilbao, Spain; ⊥ IKERBASQUE, Basque Foundation for Science, 48009 Bilbao, Spain

## Abstract

Lanthanide-based complexes with high spin are fascinating candidates for realizing spin qubits thanks to their intrinsic multilevel structure, which offers a larger number of accessible states for storing and processing information. Among the 4f series, Gd^III^, presenting an almost negligible anisotropy and *S* = 7/2, is particularly interesting for this purpose. Herein, we report on the Gd^III^-based compound [ZnCl­(μ-L)­Gd­(μ-L)­ClZn]­PF_6_ (H_2_L = *N*,*N*′-dimethyl-*N*,*N*′-bis­(2-hydroxy-3-formyl-5-bromobenzyl)­ethylene-diamine), which displays slow relaxation of the magnetization and behaves as a qubit, thereby expanding the still scarce catalogue of Gd-based molecular systems exhibiting both properties. The potential of this system for quantum computing has been explored through Q-band pulsed electron paramagnetic resonance on different diamagnetically diluted derivatives, revealing spin–lattice (*T*_1_) and phase memory (*T*_m_) times up to 249.3 and 4.938 μs at 5 K, respectively, and Rabi oscillations persisting up to 120 K.

## Graphical abstract

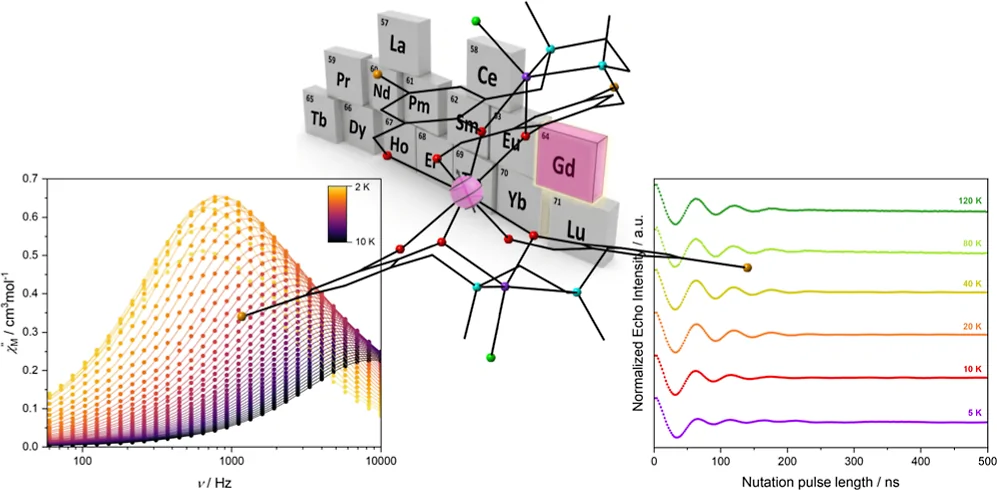

## Introduction

Since the first complex exhibiting slow relaxation of magnetization,^1^ a progressive research has been developed in the field of single-molecule magnets (SMMs),^2,3^ encouraged not only by their particular application for constructing high-density information storage devices,^4^ but also for their potential within the field of molecule-based spintronic devices^5,6^ and more recently quantum computing.^7,8^ Molecular magnetic materials are very promising candidates for encoding the building blocks of future quantum computers, i.e. qubits, and present an attractive approach that has the potential to solve some of the main limitations of the current alternative architectures (i.e., long coherence times and scalability), mainly due to the versatility, tunability and endless design possibilities provided by molecular chemistry. Among the molecular systems proposed as candidates for quantum computing, the ones based on *S* = 1/2 have attracted significant interest, because their single unpaired electron minimizes internal sources of decoherence, leading to relatively long phase memory times, as well as facilitating the understanding of their relaxation dynamics.^9^ However, their main drawback is that they can only form one-bit gates, which has prompted research toward more complex systems. In fact, the appropriate engineering of the multiple internal states of lanthanides has emerged as a promising strategy to address this limitation, as it allows to integrate multiple addressable states in a single ion, facilitating the transition from qubits, with 2 accessible states, to qudits, with d accessible states.^10,11^ Comparatively, qudits offer a larger number of accessible states for storing and processing information than that of qubits. This can lead to a simplification of the experimental setup, a reduction in circuit complexity and improved algorithm efficiency,^12,13^ allowing to encode quantum error correction protocols, a critical requirement to protect a quantum state against decoherence.^10^

In this sense, Gd^III^-based coordination complexes are ideal candidates for the realization of qudits, because, as a free ion, Gd^III^ has an isotropic electronic ground state with *L* = 0 and *S* = 7/2, the highest spin found in the periodic table. Its magnetic anisotropy then arises from weak crystal field effects mediated by spin–orbit coupling through mixing of the ground state with excited states possessing nonzero orbital angular momentum. Therefore, the chemical engineering of their first coordination sphere allows control of the energy level splitting and, consequently, the spin dynamics (note that in addition to the immediate coordination sphere, many other factors, like dipolar or intermolecular interactions, can contribute to spin dynamics).^14^ Such a magnetic anisotropy suffices to experimentally address each transition, connecting any two arbitrary states, regardless to the others. A further advantage of the Gd^III^ ion that makes it of particular interest for qubit studies is that Gd-based complexes usually exhibit small zero-field splittings, generally of various orders of magnitude smaller than those found for other Ln-based systems, for which, in some cases, only the lowest-lying electronic doublet is experimentally accessible.^15,16^

Despite the extensive work carried out over the last decades motivated by the need to find general criteria for the construction of molecular complexes behaving as both SMMs and qubits, a definitive strategy for their rational design has not been elucidated yet. In this regard, we recently studied the spin dynamics of two mononuclear gadolinium-substituted polyoxometalate-organic hybrids based on the [α-XW_11_O_39_]^8–^ (X = Si, Ge) monolacunary Keggin-type anion and the compartmental organic ligand *N*,*N*′-dimethyl-*N*,*N*′-bis­(2-hydroxy-3-formyl-5-bromobenzyl)­ethylene-diamine (H_2_L), with the general formula K_5_[Gd­(α-XW_11_O_39_)­(H_2_L)]·14H_2_O (1_X_-Gd; X = Si, Ge).^17^ The coordination of the Gd^III^ ion to the four oxygen atoms of the polyoxometalate fragment and the O_4_ external pocket of the organic ligand results in a GdO_8_ coordination polyhedra with a biaugmented trigonal prismatic (BTPR) geometry, distorted toward square antiprismatic (SAPR). When operating at Q-band frequencies, the 1_X_-Gd family behaves as a qubit up to 20 K, exhibiting spin–lattice and phase memory times up to 2315 and 2.6 μs at 3 K, respectively, for a 1% diamagnetically diluted sample. Besides, both 1_Si_-Gd and 1_Ge_-Gd display slow relaxation of the magnetization, as evidenced by maxima in the out-of-phase component of the *ac* susceptibility at *H*_dc_ ≠ 0 below 6 and 8 K, respectively.

Considering that investigations undoubtedly agree that controlling the immediate coordination environment of the paramagnetic ion is one of the most relevant factors in the quest for such materials, in this work, we investigate the effect of the coordination geometry environment of an octacoordinated Gd^III^ center on its performance as a possible SMM and qubit candidate. To this end, we broaden the catalogue of Gd compounds by developing a related complex of the recently published 1_X_-Gd (X = Si, Ge) family, in which the Gd^III^ center is simultaneously coordinated by two bridging organic ligand subunits (Figure 1).

**Figure 1:**
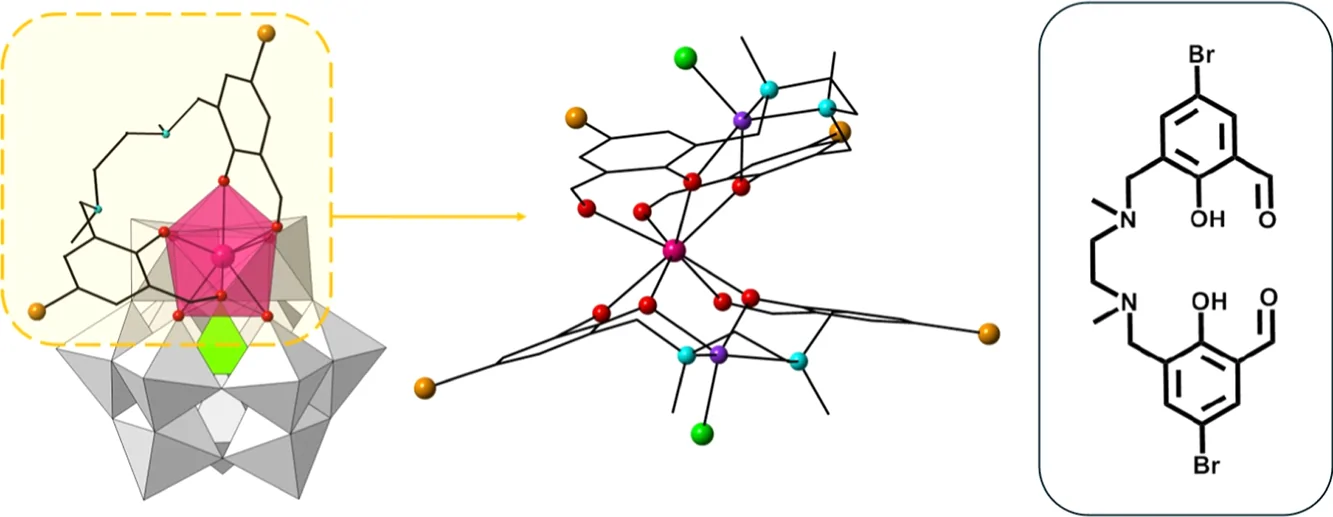
Representation of hybrid complex 1_X_-Gd (left) together with its related organic complex (middle) and the structure of the ligand (right). Color code: WO_6_, pale gray; XO_4_, green; Gd, pink; C, black; O, red; N, light blue; Br, orange; Zn, purple; Cl, light green.

Herein, we report on the synthesis and magnetic properties of the heterometallic and trinuclear Gd-based compound with the general formula [ZnCl­(μ-L)­Gd­(μ-L)­ClZn]­PF_6_. Theoretical and electron paramagnetic resonance (EPR) studies were additionally conducted for the magnetically diluted derivatives to evaluate its performance as a potential SMM and qubit candidate.

## Experimental Section

### Materials and Methods

The *N*,*N*′-dimethyl-*N*,*N*′-bis­(2-hydroxy-3-formyl-5-bromobenzyl)­ethylenediamine (H_2_L) ligand^18^ was synthesized following a reported procedure and afterward identified by means of Fourier transformed infrared (FT-IR) spectroscopy and ^1^H nuclear magnetic resonance (^1^H NMR). All chemicals were obtained from commercial sources and used without further purification. The C, H and N content of all the compounds was determined on a PerkinElmer 2400 CHN analyzer, employing around 5 mg of powdered crystalline samples. FT-IR spectra were recorded in the 400–4000 cm^–1^ region (4 cm^–1^ resolution) as KBr (Aldrich, FT-IR grade) pellets on a Shimadzu FTIR-8400S spectrometer. ^1^H NMR data for the H_2_L ligand were acquired on a Bruker ADVANCE 500 spectrometer. The sample was prepared by dissolving the organic ligand in CDCl_3_ and (CD_3_)_2_SO. Powder X-ray diffraction (PXRD) patterns were collected from 2θ = 5° to 50° (0.03° step size, 30 s per step) using a Philips X’PERT PRO diffractometer operating at 40 kV/40 mA in θ–θ configuration with monochromated Cu Kα radiation (λ = 1.5418 Å) and a PIXcel detector. Simulations of the theoretical PXRD patterns from single-crystal X-ray diffraction data were carried out using the Mercury software.^19^ The actual molar fraction of Gd^III^ and Y^III^ were determined using a Q-ICP-MS ThermoXSeries II analyzer inductively coupled plasma-mass spectrometry device. Samples were prepared by dissolving 10 mg of single crystals (from the same crystallization batch as the ones used for the EPR measurements) in an aqueous solution containing 2% of nitric acid.

### General Synthetic Procedure

The **[ZnCl­(μ-L)­Gd­(μ-L)­ClZn]­PF_6_(1-Gd)** complex was prepared by adaptation of a previously reported method.^20^ H_2_L (64 mg, 0.125 mmol), Gd­(NO_3_)_3_·6H_2_O (28.2 mg, 0.0625 mmol) and KPF_6_ (23 mg, 0.125 mmol) were added to a solution of ZnCl_2_ (8.5 mg, 0.0625 mmol) in methanol (5 mL) with continuous stirring. The mixture was left under stirring at room temperature until a yellow powder was formed. The resulting precipitate was filtered off and left to evaporate at room temperature in an open container. Yellow single crystals of **1-Gd** were obtained from the resulting clear solutions after 1 week. Yield single crystals 7%. FT-IR (cm^–1^): 1643.08 (vs), 1552.44 (s), 1452.16 (m), 1384.66 (s), 1342.23 (w), 1294.02 (s), 1216.88 (m), 1087.67 (w), 1029.82 (m), 925.68 (w), 836.97 (vs), 748.26 (m), 705.83 (m), 640.26 (m). Elem. Anal. Calcd (%) for C_40_H_40_Br_4_Cl_2_F_6_GdN_4_O_8_PZn_2_: C, 31.33%; H, 2.63%; N, 3.65%. Found: C, 30.46%; H, 2.61%; N, 4.28%.

A similar procedure was used for the synthesis of the magnetically diluted samples.

#### [ZnCl­(μ-L)­Y­(μ-L)­ClZn]­PF_6_ (**1-Y**)

Y­(NO_3_)_3_·6H_2_O (23.93 mg, 0.0625 mmol) was used as the metal source. Yield single crystals: 6%. FT-IR (cm^–1^): 1643.09 (vs), 1552.11 (s), 1451.98 (m), 1384.50 (s), 1342.21 (w), 1293.97 (s), 1217.01 (m), 1087.52 (w), 1029.85 (m), 925.73 (w), 837.00 (vs), 748.29 (m), 705.74 (m), 640.30 (m). Elem. Anal. Calcd (%) for C_40_H_40_Br_4_Cl_2_F_6_YN_4_O_8_PZn_2_: C, 32.91%; H, 2.76%; N, 3.84%. Found: C, 31.46%; H, 2.13%; N, 3.74%.

#### [ZnCl­(μ-L)­Gd_0.05_Y_0.95_(μ-L)­ClZn]­PF_6_ (**1-Gd_5%**)

Y­(NO_3_)_3_·6H_2_O (22.74 mg, 0.0594 mmol) and Gd­(NO_3_)_3_·6H_2_O (1.41 mg, 0.0031 mmol) were used as the metal source. Yield single crystals: 4%. FT-IR (cm^–1^): 1642.89 (vs), 1552.32 (s), 1452.02 (m), 1386.50 (s), 1341.98 (w), 1293.68 (s), 1216.80 (m), 1087.62 (w), 1029.80 (m), 925.43 (w), 836.84 (vs), 748.15 (m), 705.81 (m), 640.34 (m). Elem. Anal. Calcd (%) for C_40_H_40_Br_4_Cl_2_F_6_Gd_0.05_Y_0.95_N_4_O_8_PZn_2_: C, 32.83%; H, 2.76%; N, 3.83%. Found: C, 33.38%; H, 2.65%; N, 3.98%. ICP analysis afforded a Gd/Y ratio of 0.048:0.952.

#### [ZnCl­(μ-L)­Gd_0.01_Y_0.99_(μ-L)­ClZn]­PF_6_ (**1-Gd_1%**)

Y­(NO_3_)_3_·6H_2_O (23.70 mg, 0.0619 mmol) and Gd­(NO_3_)_3_·6H_2_O (0.28 mg, 0.0006 mmol) were used as the metal source. Yield single crystals: 5%. FT-IR (cm^–1^): 1642.79 (vs), 1552.04 (s), 1452.16 (m), 1385.06 (s), 1342.07 (w), 1292.62 (s), 1217.18 (m), 1087.24 (w), 1029.25 (m), 925.71 (w), 837.27 (vs), 748.16 (m), 705.83 (m), 640.03 (m). Elem. Anal. Calcd (%) for C_40_H_40_Br_4_Cl_2_F_6_Gd_0.01_Y_0.99_N_4_O_8_PZn_2_: C, 32.89%; H, 2.76%; N, 3.84%. Found: C, 33.23%; H, 3.13%; N, 4.51%. ICP analysis afforded a Gd/Y ratio of 0.013:0.987.

#### [ZnCl­(μ-L)­Gd_0.005_Y_0.995_(μ-L)­ClZn]­PF_6_ (**1-Gd_0.5%**)

Y­(NO_3_)_3_·6H_2_O (23.82 mg, 0.0622 mmol) and Gd­(NO_3_)_3_·6H_2_O (0.14 mg, 0.0003) were used as the metal source. Yield single crystals: 5%. FT-IR (cm^–1^): 1643.27 (vs), 1552.33 (s), 1452.15 (m), 1384.25 (s), 1341.84 (w), 1294.15 (s), 1216.74 (m), 1086.52 (w), 1030.84 (m), 925.02 (w), 836.53 (vs), 748.14 (m), 706.24 (m), 639.52 (m). Elem. Anal. Calcd (%) for C_40_H_40_Br_4_Cl_2_F_6_Gd_0.005_Y_0.995_N_4_O_8_PZn_2_: C, 32.90%; H, 2.76%; N, 3.84%. Found: C, 33.26%; H, 3.09%; N, 3.96%. ICP analysis afforded a Gd/Y ratio of 0.005:0.995.

### Single Crystal X-ray Diffraction

Crystallographic data for compounds **1-Gd** and **1-Y** are provided in Table S1. Intensity data were collected at 170.00(10) K on an Agilent Technologies SuperNova diffractometer equipped with monochromated Mo Kα radiation (λ = 0.71073 Å) and a HyPix (hybrid pixel array) detector for all compounds. Data frames (unit cell determination, Gaussian absorption correction with face indexing, intensity data integration, and correction for Lorentz and polarization effects) were processed using CrysAlis Pro software package.^21^ The structures were solved using OLEX2 ^22^ program and refined by full-matrix least-squares with SHELXH-1997.^23^ Final geometrical calculations were carried out with PLATON^24^ as integrated in WinGX^25^ and visualization of the structures was performed with Crystal-Maker.^26^

Thermal vibrations for all non-H atoms were treated anisotropically. Hydrogen atoms of the organic H_2_L ligands were placed in calculated positions and refined using SHELXL parameters. The anisotropic thermal ellipsoid from one of the F atoms of the counteranion in **1-Gd** was restrained using ISOR-type command from SHELXL. Continuous shape measurements (CShM) calculations were performed using the SHAPE program.^27^

### EPR Spectroscopy

CW-EPR spectra for compounds **1-Gd** and **1-Gd_5%** were recorded operating at Q-band (34 GHz) with a Bruker EMX spectrometer equipped with an ER-510-QT resonator. Pulsed EPR measurements for **1-Gd_5%**, **1-Gd_1%** and **1-Gd_0.5%** were collected on a Bruker ElexSys E580 spectrometer equipped with an ER 5106QT-2 resonator (Bruker) and a model 177ka 300 W TWT amplifier (Applied Systems Engineering Inc.). The pulses were generated using the stripline pulse-forming units (SPFUs) of the pulse microwave bridge. The sample temperature was set using a closed-circuit cryostat (Cryogenic Ltd.) controlled by a model 350 temperature controller (Lakeshore Cryotronics Inc.). Measurements were recorded at Q-band (34 GHz) and conducted on powdered crystalline samples due to the small size of the single crystals. For accuracy, tube size and position in the sample holder were kept constant during the experiments. Samples were loaded into 2 mm ID, 3 mm OD quartz tubes (Ilmasil Quartz Glass).

### *Ab Initio* Calculations

Electronic structure calculations were performed on the X-ray crystal structure of the [ZnCl­(μ-L)­Gd­(μ-L)­ClZn]^+^ cation using the ORCA 6.1.0 program package.^28^ These calculations were carried out at the complete active space self-consistent field (CASSCF) level employing the state-averaged (SA) approach. The active space consisted of the seven 4f electrons in the seven 4f orbitals of the Gd^III^ ion. One octet (*S* = 7/2), 48 sextets (*S* = 5/2), and 188 doublets (*S* = 1/2) were included in the state-average procedure. Spin–orbit coupling (SOC) was subsequently computed using the SA-CASSCF wave functions. Scalar relativistic effects were considered using the second order Douglas–Kroll–Hess (DKH) Hamiltonian. The SARC2-DKH-def2-QZVP basis set was employed for Gd, whereas the DKH-def2-TZVP basis set was used for Zn, Br, and Cl atoms, and DKH-def2-SVP for O, N, and H atoms. The calculations employed the resolution-of-identity (RI) approximation. Finally, the local magnetic properties of the Gd^III^ site were computed using the SINGLE_ANISO module as implemented in ORCA.

### Magnetic Properties

Variable-temperature magnetic susceptibility measurements were performed in the 2–300 K range using a Quantum Design MPMS3 SQUID magnetometer, under an applied field of 0.1 T. Diamagnetic corrections were estimated from the Pascal’s constants. Magnetization measurements (performed at 2 K and magnetic fields from 0 to 70 kOe) as well as alternating current (*ac*) susceptibility measurements were carried out with a PPMS (Physical Property Measurement System)—Quantum Design model 6000 magnetometer. The latter were measured in the 2–10 K range with an oscillating magnetic field of 3.5 Oe under different applied static fields and *ac* frequencies ranging from 60 to 10,000 Hz. The optimum external field of 1500 Oe was determined from field-dependent measurements performed at 2 K with an oscillating magnetic field of 3.5 Oe under different applied static fields and *ac* frequencies ranging from 60 to 10,000 Hz.

## Results and Discussion

### Crystal Structure

Reaction of Gd^III^ ions with the compartmental organic ligand *N*,*N*′-dimethyl-*N*,*N*′-bis­(2-hydroxy-3-formyl-5-bromobenzyl)­ethylene-diamine, KPF_6_ and ZnCl_2_, leads to the formation of well-shaped yellow prismatic crystals with the general formula [ZnCl­(μ-L)­Gd­(μ-L)­ClZn]­PF_6_ (H_2_L = C_20_H_22_Br_2_N_2_O_4_) (see Supporting Information for the experimental details). This polynuclear compound crystallizes in the monoclinic *C*2/*c* space group and consists of trinuclear [ZnCl­(μ-L)­Gd­(μ-L)­ClZn]^+^ complex cations and PF_6_^–^ anions (Figure S1). Within the trinuclear unit, the Gd^III^ ion is octa-coordinated to the two [ZnClL]^−^ subunits through the two phenoxy (O_p_) and the two aldehyde (O_a_) groups belonging to the compartmental ligand, with Gd–O_p_ bond distances of 2.346(3) and 2.315(3) Å and Gd–O_a_ bond distances of 2.410(4) Å (Table S2). This sandwich-type structure makes the aromatic rings of the ligand be in a quasi-coplanar conformation, with a dihedral angle of 152.19° and centroid–centroid (C_g_···C_g_) distances of ca. 7.9 Å. Besides, the trinuclear unit consists of two symmetry-related halves connected by a *C*_2_ axis passing through the Gd and P atoms of the PF_6_^–^ counteranion (Figure S1). The shortest Gd···Gd distance between neighboring [ZnCl­(μ-L)­Gd­(μ-L)­ClZn]^+^ trinuclear cations in **1-Gd** is 9.228 Å (Table S2), which is slightly longer than the intermolecular Gd···Gd distances found in complexes 1_X_-Gd at ca. >8 Å.^17^

Continuous shape measurements (CShM) performed with the SHAPE software^27^ in order to evaluate the degree of distortion of the GdO_8_ coordination sphere with respect to some eight-coordinated reference polyhedra, revealed that the Gd coordination polyhedron exhibits a CShM value of 0.617 when compared to the ideal square antiprism (SAPR, *D*_4*d*_) geometry as reference shape. CShM for other eight-coordinated reference polyhedra affords higher values (above 2.8; Table S3). These CShM values indicate that the coordination polyhedron of Gd ions can be best described as a square antiprism slightly distorted from ideality (Figure S2). This type of geometry arises from the tetradentate-O_4_ mode adopted by the compartmental ligands, allowing the incorporation of one Zn cation in their inner N_2_O_2_ pocket, which exhibits a pentacoordinate conformation that has been analyzed through CShM calculations (Table S4). Comparison with square pyramid (SPY-5, *C*_4*v*_) reference polyhedron leads to the lowest CShM value (0.598), while comparison with other penta-coordinated reference polyhedra results in significantly higher values (above 3.4). Thus, the Zn^II^ coordination sphere can be best described as a slightly distorted square pyramid and it is defined by the two O_p_ and two N atoms occupying the basal positions (with Zn–O_p_ bond distances of 2.083(3) and 2.131(3) Å, and Zn–N bond distances of 2.109(4) and 2.150(4) Å), whereas a Cl atom is found to occupy the axial position of the polyhedron (with Zn–Cl distance of 2.209(1) Å; Table S2). The adopted conformation makes the two hydrogen atoms belonging to the two aldehyde groups of the H_2_L organic ligand the closest nonzero nuclear spins to Gd^III^ ions in **1-Gd**, at a distance of 4.17 Å (Figure S3 and Table S2). This chemical feature is of high interest since interactions with nuclear spins are one of the most detrimental sources of decoherence in molecular spin systems, thus, reducing as far as possible the number of spin-active nuclei in the vicinity of the electronic-spin center becomes a crucial step to efficiently protect it against undesired interactions with its environment.

Powder X-ray diffraction measurements for a representative sample of freshly filtered crystals of **1-Gd** were conducted to examine its phase purity. The collected pattern (Figure S4) compares well with that simulated from single-crystal X-ray diffraction data, suggesting that sample **1-Gd** is constituted by a single crystalline phase.

### Continuous Wave (CW) EPR and *Ab Initio* Calculations

Continuous wave EPR spectroscopy operating at Q-band was employed to study the electronic structure of **1-Gd** (Figure S6). As is commonly found for gadolinium-based compounds, its spectrum can be easily detected at room temperature. The first-derivative spectrum is characterized by a group of broad bands centered at *g* ∼2. Considering the crystal field effects in the magnetic behavior of **1-Gd**, the spin Hamiltonian for a Gd^III^ ion is given by

1 $$Ĥ=\mathrm{g̿}\mu_{B}\beta \mathrm{B⃗}Ŝ+D\left[{Ŝ}_{Z}^{2}-\frac{1}{3}S\left(S+1\right)+E\left({Ŝ}_{X}^{2}-{Ŝ}_{Y}^{2}\right)\right]$$

The first term stands for the Zeeman splitting of the electron spin, where g̿ is the *g*-tensor, μ_B_ the Bohr magneton, *B⃗* the applied magnetic field, and *Ŝ* the spin operator. The second term is related to the second-order crystal field parameters (CFPs) *B*_2_^0^ and *B*_2_^2^ via the dependence *D* = 3*B*_2_^0^ and *E* = *B*_2_^2^, where *D* and *E* are the axial and rhombic zero-field splitting parameters, respectively. Attempts to fit the spectrum using the EasySpin toolbox based on Matlab,^29^ did not result in a satisfactory agreement over the entire spectral range, most likely due to the limited spectral resolution arising from dipolar interactions and the consequent broadening of the EPR transitions. To minimize these effects, the spectrum of a Y-diluted sample containing 5% Gd (**1-Gd_5%**) was also recorded (Figure S7). Although a satisfactory fit over the entire spectral range could not be achieved, good agreement was obtained when fitting separately the central region and the spectral wings. The fit to the central region yielded |*D*| = 0.0484 cm^–1^, |*E*| = 0.0125 cm^–1^, *g*_*x*_ = 1.994, *g*_*y*_ = 1.990 and *g*_*z*_ = 1.993 (Fit 1 in Figure S7), whereas the fit to the spectral wings yielded |*D*| = 0.0537 cm^–1^, |*E*| = 0.0149 cm^–1^, *g*_*x*_ = 1.990, *g*_*y*_ = 1.991 and *g*_*z*_ = 1.993 (Fit 2 in Figure S7). The difference between the two fits indicates that the *D* and *E* parameters cannot be determined uniquely from the present data; nevertheless, their actual values are expected to fall within the range defined by the two fitting approaches. It should be noted that the axial and rhombic zero-field splitting (ZFS) parameters are reported as absolute values, since their signs cannot be determined from room-temperature measurements owing to the negligible thermal dependence of the relative transition intensities. The values obtained are in good agreement with those reported in the literature for related Gd^III^-based complexes.^15,30^

Alternatively, *ab initio* calculations performed on the [ZnCl­(μ-L)­Gd­(μ-L)­ClZn]^+^ cation using the Orca software^28^ and based on the complete active space self-consistent field (CASSCF) approach, afforded *D* and *E* parameters of 0.0262 and −0.0065 cm^–1^, respectively (Table S5). Such calculated terms compare well with the parameters obtained from experimental Q-band CW EPR spectra. Both approaches indicate a significant rhombic contribution to the magnetic anisotropy, as reflected by the large |*E*/*D*| ratio of ca. ∼0.25–0.28. The *ab initio* calculations suggest that this rhombicity originates predominantly from the nonaxial crystal-field parameter *B*_2_^2^, the magnitude of which largely exceeds that of the remaining CFPs (Table S6). Furthermore, both experimental and *ab initio* results indicate that the low-lying Kramers doublets span a narrow energy range of less than 1 cm^–1^. This energy scale is comparable to the microwave frequency accessible by conventional EPR spectroscopy, suggesting the possibility of addressing the transitions between these states using microwave resonant pulses (Figure S8).

### Magnetic Properties

*Direct-current* (*dc*) magnetic susceptibility measurements for complex **1-Gd** were carried out on a randomly oriented polycrystalline sample in the 2–300 K temperature range under an applied magnetic field of 0.1 T, as depicted in Figure S9. The experimental χ_M_ *T* value of 7.86 cm^3^ K mol^–1^ at 300 K for **1-Gd** is in good agreement with the calculated value of 7.88 cm^3^ K mol^–1^ for a free Gd ion with ^8^ *S*_7/2_ and *g*_J_ = 2. Upon cooling, the experimental χ_M_ *T* product remains almost constant until 13 K, as expected from the isotropic nature of Gd^III^ ions. Below this temperature, χ_M_ *T* goes down sharply, reaching a value of 7.23 cm^3^ K mol^–1^ at 2 K. For such an ion with no expected orbital angular momentum contributing to its ^8^ *S*_7/2_ ground state, the χ_M_ *T* product is supposed to remain constant throughout the whole range of temperature. The observed deviation at low temperature toward smaller values suggests weak intermolecular antiferromagnetic interactions, dipolar coupling between neighboring molecules and/or weak anisotropy effects.

Field-dependent magnetization measurements in the presence of a magnetic field μ_0_ *H* = 0–7 T at 2 K (Figure S10) reveal a rapid increase in the magnetization with the applied field, which practically saturates at 3 T, reaching a value of 6.99 μ_B_ for a 7 T field, in good agreement with the theoretical saturation value of ca. 7 μ_B_. The experimental magnetic susceptibility and magnetization data are in good agreement with the corresponding theoretical curves simulated using the PHI program^31^ with the |*D*| and |*E*| values deduced from the CW-EPR spectra, i.e. 0.0484 and 0.0125 cm^–1^, respectively.

In order to study if the synthesized complex exhibits slow relaxation of the magnetization, *alternating-current* (*ac*) magnetic susceptibility measurements were performed as a function of both temperature and frequency on a microcrystalline sample of **1-Gd**. At zero applied magnetic field, no contribution to neither the in-phase (χ_M_^′^) nor the out-of-phase (χ_M_^″^) signals was observed. However, in the presence of an external magnetic field, well-marked maxima of the out-of-phase signals are clearly visible (Figure S11). Scans of the out-of-phase magnetic susceptibility with varying the magnetic field at a fixed temperature of 2 K, revealed an optimal magnetic *dc* field of 1500 Oe for **1-Gd**, where a single maximum with the slowest relaxation time is obtained (Figures S11 and S12). The *ac* magnetic susceptibility of complex **1-Gd** at the optimum field exhibits temperature-dependent peaks that gradually shift to lower frequencies as the temperature decreases, observed up to 10 K at frequencies ≥550 Hz, implying the occurrence of slow relaxation of the magnetization under these experimental conditions (Figure 2).

**Figure 2:**
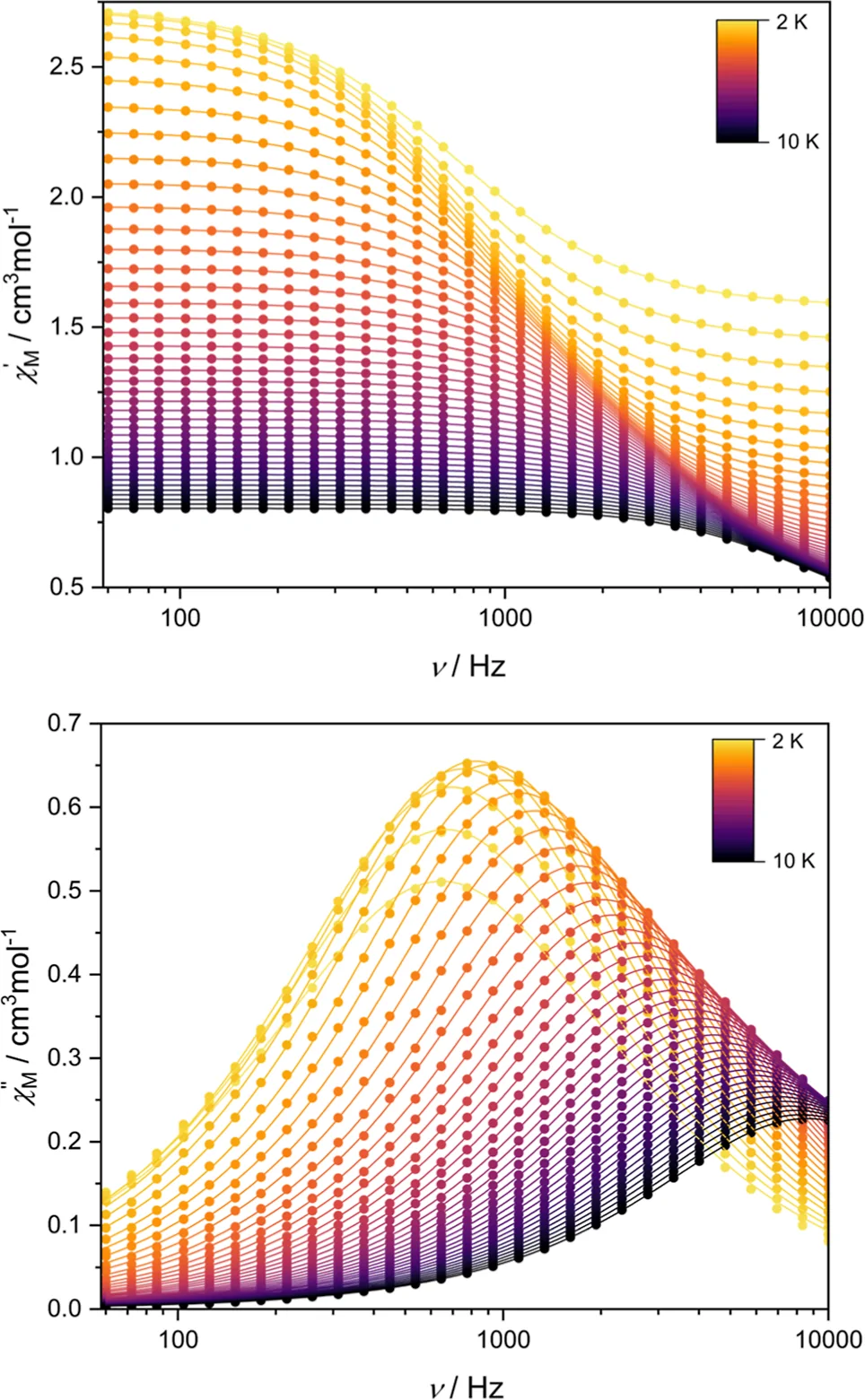
(Top) Frequency dependence of the in-phase (χ_M_^′^) and (bottom) the out-of-phase (χ_M_^″^) component of the *ac* susceptibility at *H*_dc_ = 1500 Oe for **1-Gd**. The experimental data are denoted by circles; solid lines represent the best fit of the data.

In order to get insight into the relaxation mechanisms, the frequency-dependent in-phase and out-of-phase magnetic susceptibility data between 2 and 10 K were fitted to the generalized Debye model (Figure 2 and Table S7), which allowed to extract the temperature dependence of the characteristic relaxation times (Figure S13). Inspection of the Cole–Cole plot (Figure S14) reveals α values between 0.013 and 0.085 in the studied temperature range (Table S7), indicating a relatively narrow distribution of relaxation times.

Although a linear regime is observed in the ln τ vs *T* ^–1^ plot at the highest temperature values, the curvature of the relaxation times observed at lower temperatures indicates that relaxation cannot be described by a pure Orbach process (Figure S13). Importantly, CW-EPR measurements and *ab initio* calculations reveal only a very small axial zero-field splitting, corresponding to a maximum energy separation of approximately 12|*D*| ∼ 0.6 cm^–1^ between the lowest and highest Kramers doublets. This barrier is readily overcome thermally at the temperatures investigated above 2 K and therefore cannot account for the observed slow relaxation. Consequently, the slow relaxation of the magnetization is unlikely to originate from an Orbach process involving a well-defined magnetic energy barrier.

While the data over the whole range of temperatures does not follow the Arrhenius law, it almost perfectly follows the power law described by eq 2 containing the conventional Raman term. The fitting affords *n* = 1.847, which is much lower than the one expected for a Raman mechanism (i.e., 7 or 9), but closer to those of a direct (*n* = 1) and phonon bottleneck processes (*n* = 2).

2 $$\tau^{-1}=BT^{n}$$

Although the lowest temperature data are not perfectly fitted with this phenomenological equation, it was not possible to better fit the relaxation rates using alternative terms or even considering a combination of them. Considering this, we can conclude that both mechanisms could be responsible for the slow magnetic relaxation of the magnetization observed in **1-Gd**, as already reported in the literature.^32,33^

### Pulsed EPR Experiments

With the aim of studying the spin dynamics of **1-Gd**, and thus, its viability as a candidate for quantum information processing, magnetically diluted samples of its yttrium analogue **1-Y** were prepared. Reactions were carried out under the same synthetic conditions as those employed for **1-Gd**, varying the molar fraction of Gd^III^ with respect to the total metal ion content, yielding final materials with molar percentages of Gd as low as 5% (**1-Gd_5%**), 1% (**1-Gd_1%**) and 0.5% (**1-Gd_0.5%**). These concentrations were chosen to investigate the effects of dipolar interactions contributing to dephasing.

Pulsed EPR experiments were conducted by using a few milligrams of powdered crystalline samples due to the small size of the single crystals. Starting with the most basic characterization, all diluted samples exhibit intense Q-band echo-detected field-swept (EDFS) signals that remain measurable at least up to 120 K (Figures 3, S15, and S16). The fact that an electron spin–echo is detected in the EDFS spectrum is an indication that quantum coherence is expected for this Gd-based complex. Note that the experimental EDFS spectra are in relatively good agreement with that simulated from the set of spin Hamiltonian parameters described above (Figure S17). The recorded spectra of the different diluted samples display similar spectroscopic features, with a main resonance line located at 12,184 G. On the basis of these results, inversion recovery and Hahn-echo experiments were performed at this field position. The attribution of the observed main band to a specific transition between different electronuclear states of single Gd^III^ centers is not trivial, as can be deduced from the energy diagram in the *x*-, *y*- and *z*-directions simulated from the parameters obtained from Q-band CW EPR spectroscopic experiments (Fit 1, see above). The resulting diagram allows to allocate the possible transitions, represented as red lines in Figure 3, in which the energy gap matches the frequency of the microwave pulse. Such transitions are particularly overlapped across the *x*-direction within the 12,064–12,345 G field range and are predominantly associated with the main central line observed in the experimental EDFS spectra.

**Figure 3:**
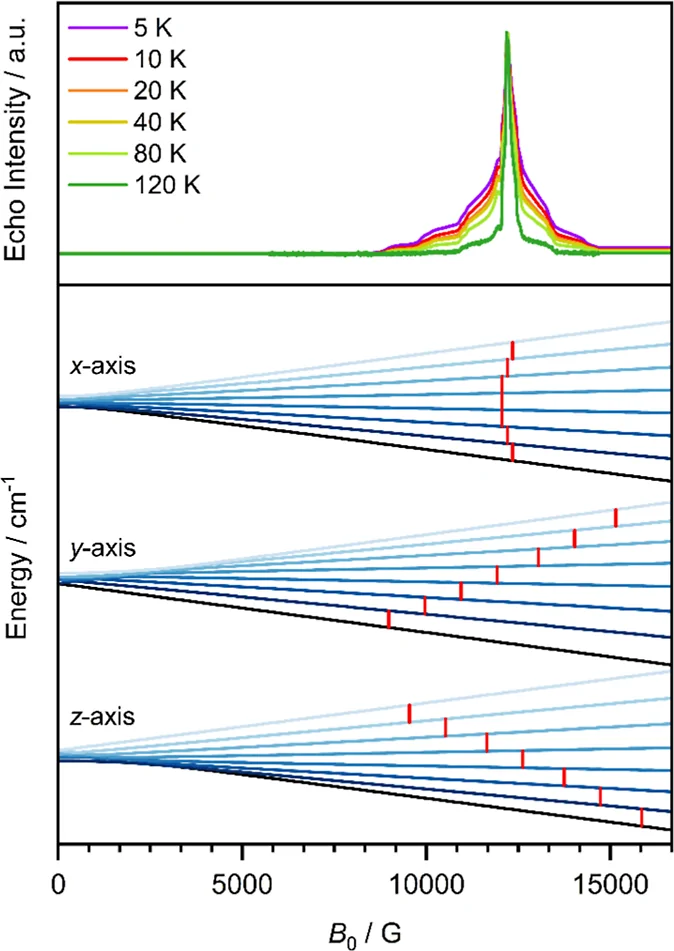
Echo-detected field-swept EPR spectra (34 GHz; π/2 pulse of 16 ns) of **1-Gd_5%** at different temperatures and simulated 8-fold energy levels of the *S* = 7/2 multiplet in *x*-, *y*- and *z*-axes as a function of the *B*_0_ magnetic field performed with the PHI program.^31^ The blue lines represent the simulation with the parameters extracted from the Q-band CW-EPR spectra and the red lines represent possible transitions in the *x*-, *y*- and *z*-axes.

Inversion recovery decay curves induced by an inversion recovery pulse sequence were obtained to elucidate the spin–lattice relaxation times, *T*_1_, as depicted in Figures S18–S20. To this end, data from the saturation recovery experiments were fitted to a biexponential model, as given in eq 3

3 $$I\left(t\right)=A\;\exp\left(-t/T_{1}\right)+B\;\exp\left(-t/T_{1.s}\right)+y_{0}$$

where the longer, *T*_1_, is assumed to be the spin–lattice relaxation time and the shorter, *T*_1.s_, the spectral diffusion time. As it is shown in Figures 4 and S18–S20, *T*_1_ times display strong temperature dependence within the tested temperature region for all diluted samples, reaching a maximum value of 249.3 μs for **1-Gd_0.5%** at 5 K, whereas for the more concentrated derivative, i.e. **1-Gd_5%**, this value drops to 156.1 μs (Figure S21 and Tables S8–S10). The decrease in *T*_1_ with increasing the spin concentration is not surprising, because stronger coupling effects between neighboring electronic spins are expected, contributing to faster relaxation. Meanwhile, at higher temperatures, spin–lattice relaxation times are nearly independent of the concentration, and the differences between them become almost negligible upon increasing the temperature, ranging from 46.2 to 60.9 μs at 10 K and decaying until 0.55 μs at the highest temperature value (i.e., 120 K). This sharp decrease observed when warming from 5 to 10 K is consistent with the presence of phonon contributions, which typically become significant at higher temperatures. The fact that the three magnetically diluted derivatives display almost identical *T*_1_ values across the 10–120 K temperature range is an indication that the phonon bath might be dominating the spin relaxation process at high temperatures, rather than spin–dipolar coupling. Whereas at very low temperatures, the latter’s contribution is notable.

**Figure 4:**
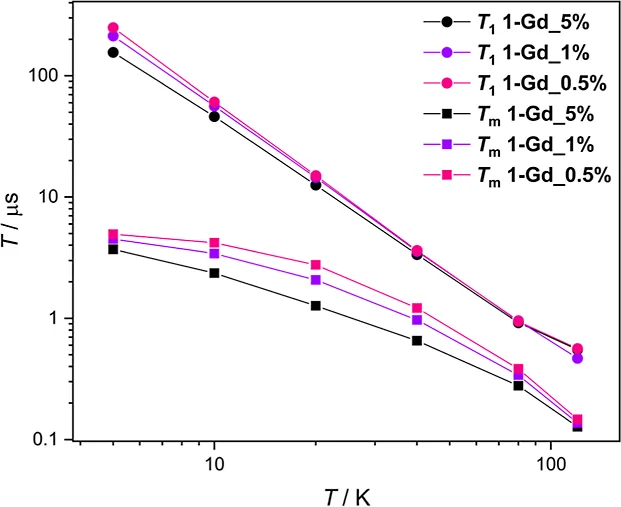
Temperature dependence of *T*_1_ (scatter) and *T*_m_ (square) for **1-Gd_5%**, **1-Gd_1%** and **1-Gd_0.5%** at *B*_0_ = 12,184 G and different temperatures. Solid lines represent a guide to the eye. Note the double logarithmic scale on both *x*- and *y*-axes.

Additionally, spin–lattice relaxation times for all diluted derivatives follow a power law described by the dependence $\frac{1}{T_{1}}=AT^{n}$ , affording an *n* value equal to 1.875(4), 1.91(2) and 1.955(4) for **1-Gd_5%**, **1-Gd_1%** and **1-Gd_0.5%**, respectively (Figure S22). It is worth noting that the experimental data acquired at 120 K deviate from the fitted trend and were therefore excluded from the analysis. This behavior may arise either from experimental errors associated with the measurements at high temperatures or from a different temperature dependence of *T*_1_ in the high temperature regime. Such a small exponent is likely related to a direct phonon relaxation process limiting *T*_1_, and is consistent with previously reported values for other Gd-based complexes found in the literature.^16,34,35^ Interestingly, both *ac* and EPR measurements yielded very similar values of the *n* exponent, even though comparison of spin relaxation times using different techniques is not straightforward due to the completely different molecular environment in which the measurements are performed.

With respect to *T*_m_, coherence times were determined from the fitting of Hahn-echo decay curves to an exponential equation containing the stretching factor β, according to

4 $$I\left(2\tau \right)=A\;\exp{\left(-2\tau /T_{m}\right)}^{\beta }+y_{0}$$

It should be noted that, as expected for all compounds, the experimental Hahn-echo signal intensities rapidly decayed with increasing the interpulse delay, 2τ, and the rate of the decay increased with the temperature (Figures S23–S25). In contrast to *T*_1_, phase-memory times have a weaker temperature dependence within the tested temperature range, especially at low temperatures up to 20 K (Figure 4). However, above this temperature, the extracted times decrease faster until reaching the lowest detectable value at 120 K. Interestingly, under these thermal conditions, *T*_m_ and *T*_1_ reach comparable values, indicating that phase memory times become limited by spin–lattice decoherence at high temperature. This trend has been previously observed in many other reported species^9,36–38^ and is consistent with the dependence *T*_2_ ≤ 2*T*_1_, which establishes the upper limit of *T*_m_.

As shown in Figure 5, comparison between phase memory time values of the three diluted derivatives at the same temperature indicates that the more diluted sample, i.e. **1-Gd_0.5%**, is more effective for *T*_m_ enhancement via suppression of dipolar Gd···Gd interactions that contribute to dephasing. As a result, **1-Gd_0.5%** exhibits phase memory times up to 4.938 μs at 5 K, while the samples with 1% and 5% Gd doping levels display maximum values of ca. 4.505 and 3.706 μs, respectively.

**Figure 5:**
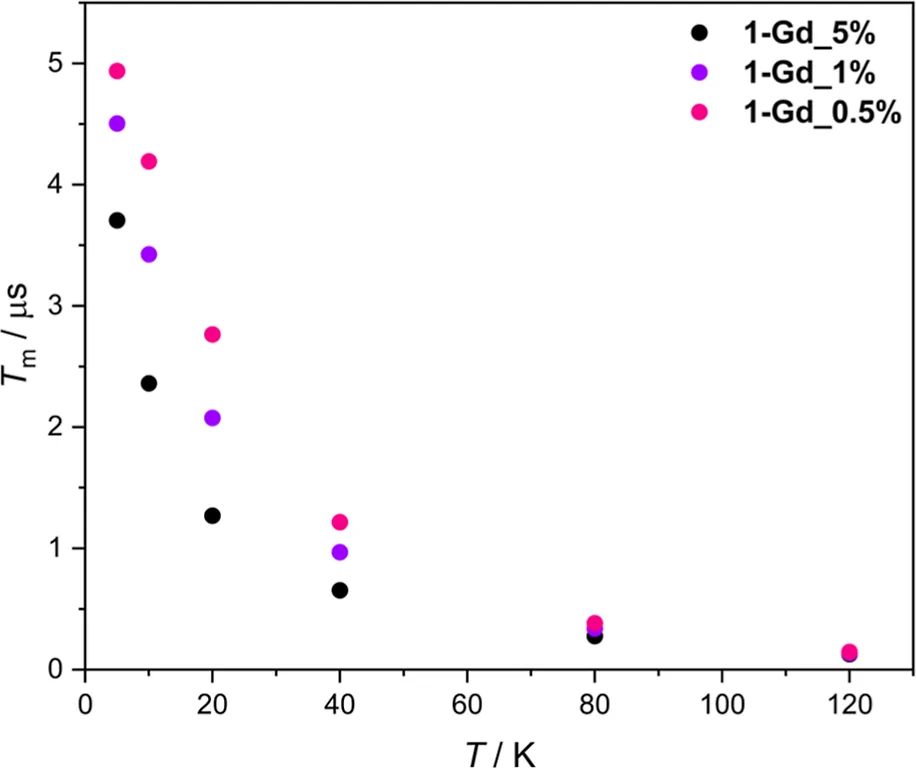
Phase memory times, *T*_m_, obtained at *B*_0_ = 12,184 G and different temperatures for **1-Gd_5%**, **1-Gd_1%** and **1-Gd_0.5%**.

It should be mentioned that the temperature dependence of the best-fit values of the stretching parameter follows a similar trend across the three magnetically diluted samples, being typically higher than 1 below 40 K and gradually tending toward a monoexponential decay as the temperature increases (Figure S26 and Tables S11–S13). Such values, slightly deviated from β = 1, suggest that physical motion of nuclear spins could be the mechanism dominating the relaxation.^39^ Moreover, this relatively wide range of values could be due to the echoes arising simultaneously from different transitions (with different relaxation times) at the selected *B*_0_ field.

Transient nutation experiments enabled the observation of coherent Rabi oscillations at a wide range of temperatures for all the magnetically diluted derivatives, proving the possibility to perform coherent manipulation of their spin states, i.e. the possibility to place the electronic spin in any arbitrary superposition of its constituent states. This oscillatory behavior was observed up to 120 K as presented in Figure S27. The Rabi frequencies (Ω_R_) of the resulting oscillations at 10 K were obtained by zero-filling these curves followed by a fast Fourier transform, which results in a prominent peak observed at each microwave power (Figure 6). As expected, the spin-flip time scale (defined as the time separating an oscillation maximum from an adjacent minimum) decreases with the strength of the pulse powers, i.e. the stronger *B*_1_ is, the higher the Rabi frequency is at a given *B*_0_ field. The identity of the Rabi frequencies is clearly demonstrated by their linear dependence on the microwave power, *B*_1_, which evidence that the origin of these oscillations can be attributed to quantum control and not to nuclear modulation or cavity background effects (Figure 6).

**Figure 6:**
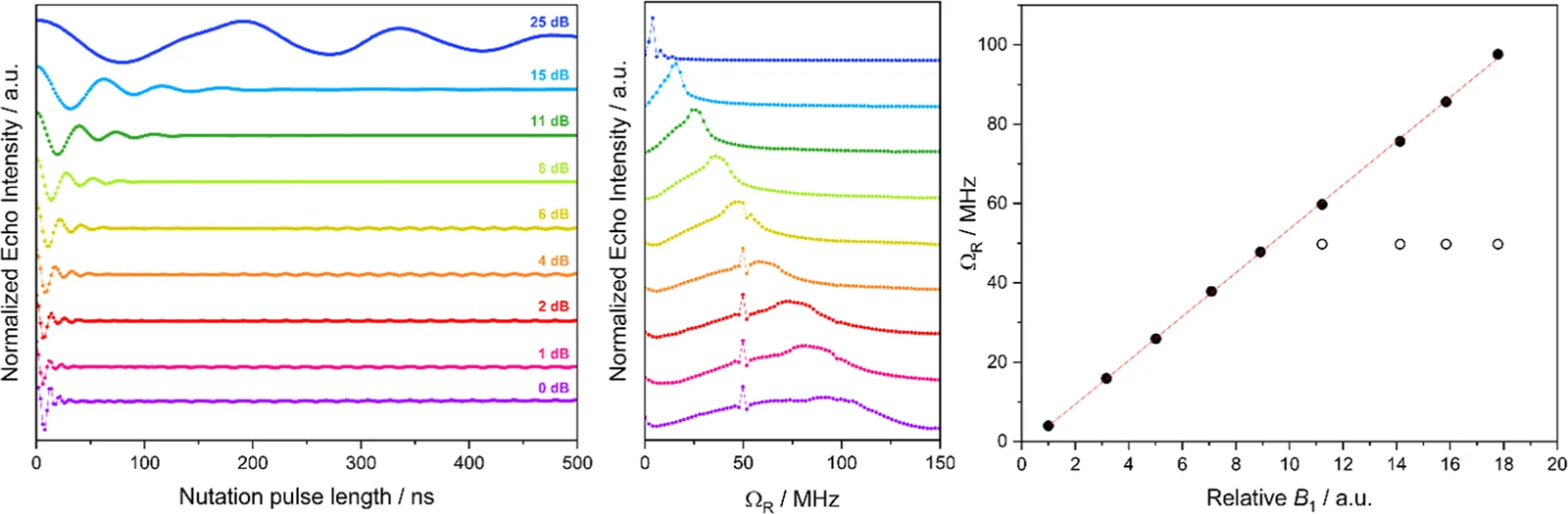
Left: Rabi oscillations recorded at 10 K and *B*_0_ = 12,184 G using different microwave powers for **1-Gd_0.5%**. Middle: their corresponding Fourier transforms. Right: *B*_1_ dependence of the Rabi frequencies (full circles), Ω_R_, and the additional high-frequency oscillations (open circles), Ω_H_, ( $B_{1}\propto \sqrt{P}$ where *P* is the microwave power). The red solid line is the linear fit of Ω_R_.

It is also noteworthy that Fourier analyses of the transient nutation data reveal some power-independent features at 49.8 MHz for several *B*_1_ values, corresponding to the Larmor frequencies of the hydrogen nuclei at the respective field value.^40^ Such observation suggests a mechanism of coherence transfer between the electronic spin of Gd^III^ and the nuclear spin of ^1^H, most probably induced by the closest hydrogen atoms of the organic ligand.

The qubit figure of merit, *Q*_M_ = 2*T*_m_Ω_R_, calculated from the experimental *T*_m_ and Ω_R_ value at the lowest microwave power, reaches up to ∼964 in complex **1-Gd_0.5%** at 5 K. Even though this value is not comparable in terms of magnitude with those found for some transition metal complexes in strictly nuclear spin-free environments^41,42^ and is still quite far from the lower threshold required for its implementation in quantum information technologies, the number of observed coherent rotations is considerably higher than those observed in other lanthanide-based metal–organic complexes.^43,44^

### Comparison with Related Gd-Based Molecular Magnets and Qubits

Compared to the previously reported 1_X_-Gd family (|*D*| = 0.0802 cm^–1^ and |*E*| = 0.0231 cm^–1^ for the Si derivative, |*D*| = 0.0831 cm^–1^ and |*E*| = 0.0244 cm^–1^ for the Ge derivative), modifications in the Gd environment of **1-Gd** lead to smaller zero-field splitting parameters. As a result, the systems exhibit different energy level diagrams.

As in the case of 1_X_-Gd, **1-Gd** displays slow relaxation of the magnetization but the frequency-dependent *ac* susceptibility signals are slightly shifted toward higher temperatures. This trend could be explained by the structural changes in the immediate coordination sphere of the Gd^III^ ion, together with the combination of diamagnetic 3d–4f ions in **1-Gd**, which have been proven to be a suitable approach to enhance the magnetic behavior of a given system.^45^ Note that from a magnetic point of view, such diamagnetic ions can be considered as magnetic dilutors and thus, the resulting 3d–4f mixed complexes can be treated as mononuclear, i.e. the magnetic properties only depend on the 4f ion.

The occurrence of slow relaxation of the magnetization in Gd^III^-based compounds is scarcely found in the literature compared to other Ln^III^-based complexes. This fact is somehow not surprising considering the isotropic nature of Gd^III^ as a free ion due to its half-occupied 4f^7^ electron configuration and its zero orbital contribution. Among the infrequently reported examples containing only one Gd^III^ ion, i.e. monometallic purely 4f complexes (Table S14) and heterometallic 3d–4f complexes with nonparamagnetic 3d ions (Table S15), this phenomenon has usually been explained by the presence of relaxation mechanisms other than Orbach. Nonetheless, relaxation dynamics dominated by this relaxation pathway have been previously reported in the Gd-containing polyoxometalate anion [Gd­(W_5_O_18_)_2_]^9–^.^44^ Commonly, Raman or direct processes are suggested to explain the appearance of such behavior, while the application of external magnetic fields can efficiently suppress, at least partially, the quantum tunneling of the magnetization (QTM) processes that are often responsible for the fast magnetic relaxation observed in Gd-based compounds.^32,46–51^ Although medium-to-high external fields (≥2000 Oe) are frequently employed in the study of Gd­(III) systems exhibiting slow magnetic relaxation, QTM suppression can in some cases be achieved at considerably lower fields. In addition, the use of relatively high fields may be related to the continuous shift of the maximum of the out-of-phase component of the *ac* susceptibility (χ″) toward higher temperatures with increasing field, rather than being solely required for the suppression of QTM. In this context, well-defined maxima in the χ″ signals under comparatively low applied fields, such as 1000 Oe, remain much less frequently reported in the literature. Furthermore, the relaxation dynamics exhibited by **1-Gd** are observed at low temperatures (up to 10 K), which is somewhat similar to other previously reported Gd^III^ systems (Tables S14 and S15).

Interestingly, to the best of our knowledge, only five of the reported systems have also been proposed as molecular qubit candidates for quantum computing, with **1-Gd** further extending this limited catalogue.^15,17,30,46^ It should be noted, however, that the observation of spin-coherence phenomena in Gd^III^ complexes is not uncommon and has been extensively demonstrated by pulsed-EPR techniques, particularly in the context of Gd^III^-based spin labels for pulse-dipolar EPR and (double electron–electron resonance) DEER measurements.^52^

The very few Gd-based systems showing both slow relaxation of the magnetization and spin-coherence phenomena, together with the diverse geometrical structures around the Gd^III^ ion, make it difficult to extract clear structural connections between molecular magnets and qubit systems. It is worth mentioning that **1-Gd** shows slightly higher *T*_m_ values than those observed for its related polyoxometalate-organic hybrid derivative.^17^ One could think that the magnetic insulation and robustness conferred by this Keggin-type polyoxoanion would enhance the coherence times of the system through suppression of nuclear spins and electron–phonon interactions. However, the use of exclusively nonrigid ligands in the purely organic derivative (i.e., **1-Gd**) affords a system with a certain degree of flexibility around the lanthanide ion. In this way, it allows the accommodation of a diamagnetic cation in the empty N_2_O_2_ inner site of the compartmental ligand, thus preventing significant deviations from planarity. This fact also promotes the deprotonation of the N atoms from the N_2_O_2_ pocket in comparison to 1_X_-Gd, which confers a nuclear spin free environment for the Gd^III^ ion. Furthermore, the use of PF_6_^–^ as counteranion results in a *C*_2_ axis passing through the Gd and P atoms of the counteranion, leading to a smaller degree of distortion of the GdO_8_ coordination polyhedron when compared to 1_X_-Gd. These changes in the immediate coordination sphere of the Gd^III^ ion would cause differences in its crystal field, influencing its magnetic properties and thus, its spin dynamics. The influence of structural changes on the electronic and magnetic properties has been previously reported in the literature and could explain the improvement in the qubit performance of **1-Gd**.^36^ Even if the studied system does not present record-breaking quantum coherence times, the calculated values are in the typical *T*_m_ range for Gd^III^-based molecular systems, as illustrated in Table S16. Nevertheless, a direct comparison between the different systems compiled in this table should be avoided, since the reported coherence values have not been obtained under the same experimental conditions. Indeed, the obtained values are still far from those reported in alternative qubit platforms (e.g., Si, SiC, N-vacancies in diamond, etc.);^53–56^ nonetheless, the modular nature of molecular qubits offer design strategies that are not available in classical purely inorganic systems.

## Conclusions

In this study, we have extensively investigated the slow relaxation of the magnetization and the potential for coherent manipulation of spin information in a polynuclear Gd^III^-substituted system by means of Q-band pulsed EPR experiments, highlighting at the same time the potential power behind the Gd chemistry to develop molecular platforms to realize viable qubits for quantum information. Although no temperature dependence at zero applied magnetic field is observed for the reported system, it exhibits slow relaxation of the magnetization under the presence of an external field at low temperatures, making **1-Gd** one of the relatively infrequent Gd^III^-based complexes exhibiting this phenomenon. Upon diamagnetic dilution with Y^III^ analogues, this compound shows phase memory and spin–lattice times up to 4.938 and 249.3 μs at 5 K, respectively, which are comparable with the ones reported for other Gd^III^ molecular systems. Such coherence time values enabled the observation of Rabi oscillations in the 5–120 K temperature range, confirming the coherent spin state control in **1-Gd**. Based on the results presented herein, together with those from previously reported studies, it has been proven that even though manipulating an *S* = 7/2 spin system is quite challenging, it is not impossible. The prospect of using such high-spin systems could be especially beneficial for enhancing information density when compared to the simplest *S* = 1/2 spin-based systems. The key lies in optimizing their coherence properties through appropriate ligand environments and coherence protection schemes.

## Supplementary Material



## Notes

Single crystal X-ray diffraction, continuous shape measurements, powder X-ray diffraction, CW-EPR spectroscopy, *ab initio* calculations, magnetic properties, pulsed EPR spectroscopy, references (PDF)

## Abbreviations

H_2_L: *N*,*N*′-dimethyl-*N*,*N*′-bis­(2-hydroxy-3-formyl-5-bromobenzyl)­ethylene-diamine
*T*_1_: spin–lattice relaxation time
*T*_m_: phase memory relaxation time
*T*_2_: spin–spin memory relaxation time
*L*: angular orbital momentum
*S*: spin
SMM: single molecule magnet
BTPR: biaugmented trigonal prism
SAPR: square antiprism
SPY-5: square pyramid
ZFS: zero-field parameters
EPR: electron paramagnetic resonance
CShM: continuous shape measurements
CW: continuous wave
*dc*: direct-current
*ac*: alternating-current
QTM: quantum tunneling of the magnetization
*U*_eff_: effective energy barrier
EDFS: echo-detected field-swept
DEER: double electron–electron resonance
