# Supplementary material for: Tuning Crystal Field and Spin Dynamics in Gd(III) Complexes via Coordination Geometry
Source: Inorg Chem. 2026 Oct 13;65(42):24786–96. doi: 10.1021/acs.inorgchem.6c03603 (PMC13715582; doi:10.1021/acs.inorgchem.6c03603)
Supplement: Supplementary file 1 [file ic6c03603_si_001.pdf]

SUPPORTING INFORMATION

Tuning crystal field and spin dynamics in Gd(III) complexes via coordination geometry

Corina Rodríguez-Esteban,[a,b,c] Daiana Cabrosi,[a] Beñat Artetxe,[c] Luis Lezama,[c] Mathieu Gonidec,[b] Itziar Oyarzabal*[a,d]

a. BCMaterials, Basque Center for Materials, Applications and Nanostructures, 48940 Leioa, Spain.
b. Univ. Bordeaux, CNRS, Bordeaux INP, ICMCB, UMR 5026, 33600 Pessac, France.
c. Departamento de Química Orgánica e Inorgánica, Facultad de Ciencia y Tecnología, Universidad del País Vasco UPV/EHU, 48080 Bilbao, Spain.
d. IKERBASQUE, Basque Foundation for Science, 48009 Bilbao, Spain.

Email: itziar.oyarzabal@bcmaterials.net

Table of Contents

Single Crystal X-Ray Diffraction 2
Continuous Shape Measurements 4
Powder X-Ray Diffraction 5
CW-EPR Spectroscopy 6
***Ab-initio* Calculations** 7
Magnetic properties 9
Pulsed EPR Spectroscopy 12
References 24

Single Crystal X-Ray Diffraction

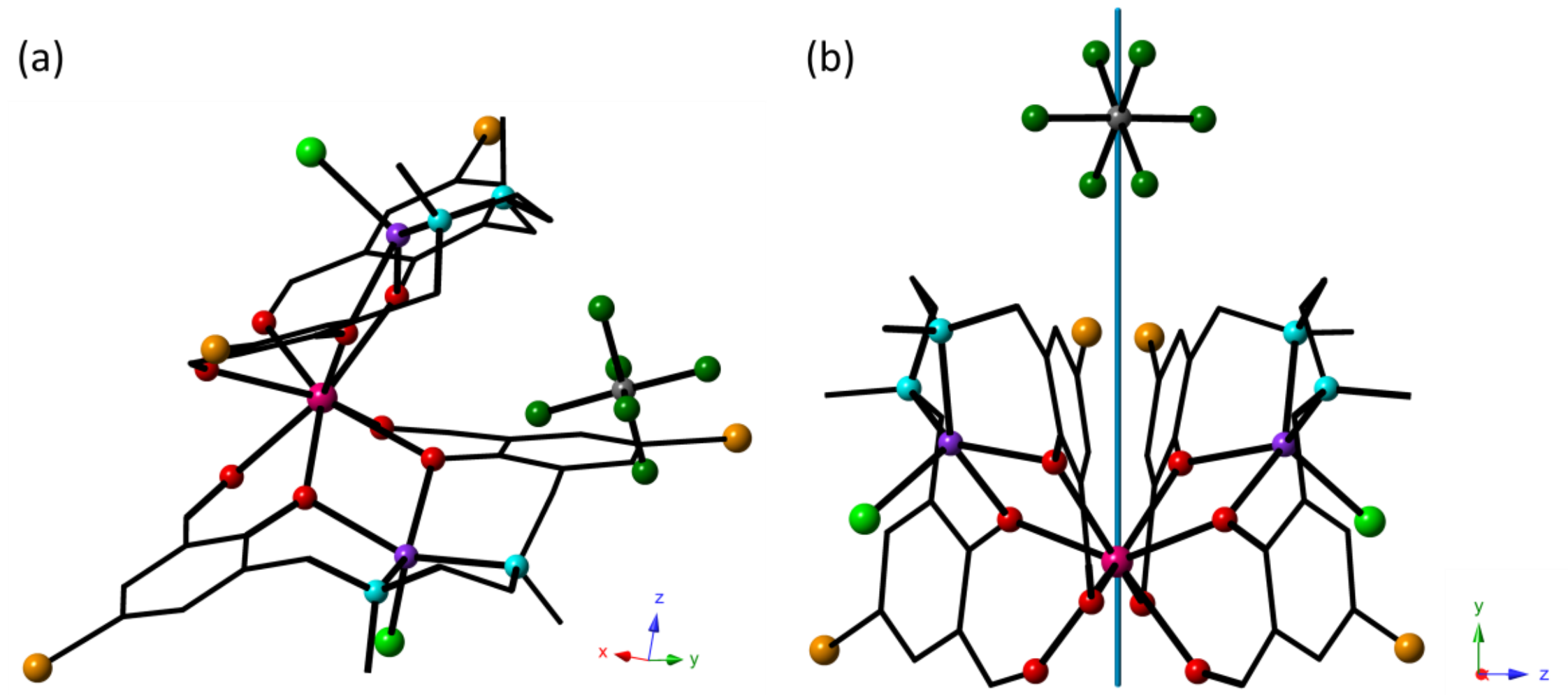

Figure S1. (a) Representation of the molecular structure of complex **1-Gd**, and (b) view of the C_2 axis passing through the Gd and P atoms along the crystallographic *x* axis. Hydrogen atoms have been omitted for clarity. Colour code: Gd, pink; C, black; O, red; N, light blue; Br, orange; Zn, purple; Cl, light green; P, grey; F, dark green.

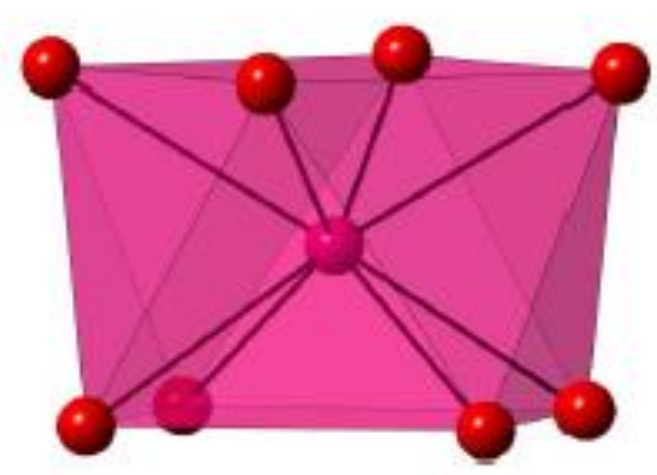

Figure S2. Representation of the square antiprism GdO8 coordination sphere in **1-Gd**. Colour code: Gd, pink; O, red.

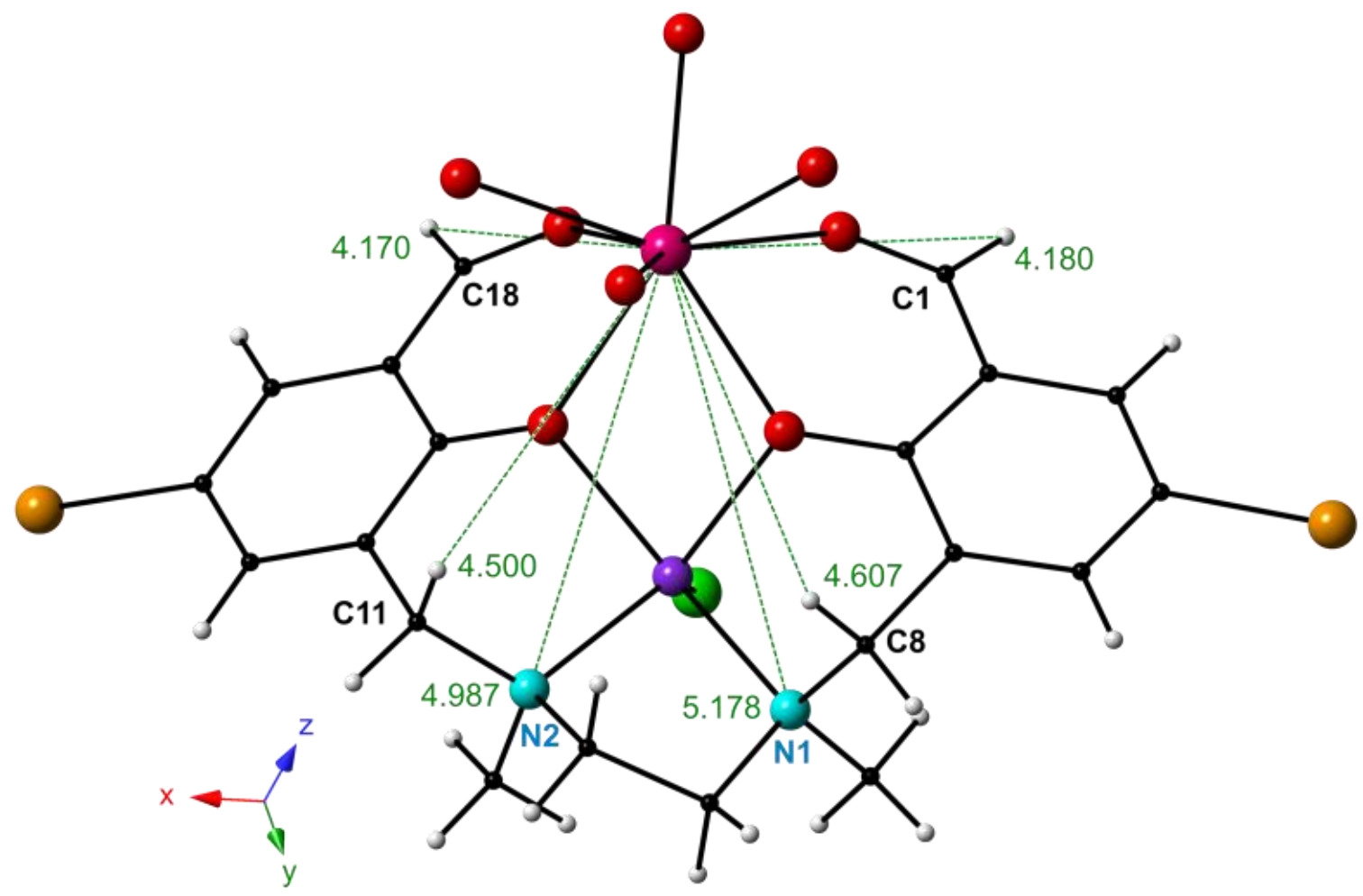

Figure S3. Selected Gd···H intramolecular distances (Å) in **1-Gd**. Colour code: Gd, pink; C, black; O, red; N, light blue; Br, orange; Zn, purple; Cl, light green; P, grey; F, dark green.

Table S1. Crystallographic data for **1-Gd** and **1-Y**.

	**1-Gd**	**1-Y**
**Empirical formula**	$C_{40}H_{40}Br_4Cl_2F_6GdN_4O_8PZn_2$	$C_{40}H_{40}Br_4Cl_2F_6YN_4O_8PZn_2$
**fw (g·mol^{-1})**	1528.26	1459.92
**Crystal system**	Monoclinic	Monoclinic
**Space group (number)**	*C2/c* (15)	*C2/c* (15)
***a* (Å)**	17.0619(7)	17.0964(4)
***b* (Å)**	18.2799(8)	18.2722(4)
***c* (Å)**	16.0431(6)	15.9922(4)
***α* (deg)**	90	90
***β* (deg)**	97.256(4)	97.644(2)
***γ* (deg)**	90	90
***V* (Å^3)**	4963.6(3)	4951.4(2)
Z	4	4
***μ* (mm^{-1})**	5.715	5.568
**Reflns collected**	18658	34025
**Unique reflns (R_{int})**	5979 (0.045)	6293 (0.036)
**Observed reflns [$I > 2\sigma(I)$]**	4035	4907
**Params. / restr.**	310 / 12	310 / 0
**$R(F)^a$ [$I > 2\sigma(I)$]**	0.045	0.041
**$wR(F^2)^b$ [all data]**	0.109	0.096
**GoF**	1.063	1.056

$^aR(F) = \sum||F_o - F_c||/\sum|F_o|$. $^bwR(F^2) = \{\sum[w(F_o^2 - F_c^2)^2]/\sum[w(F_o^2)^2]\}^{1/2}$.

Table S2. Selected bond lengths (Å), interatomic distances (Å) and angles (°) for compounds **1-Gd** and **1-Y**.

	**1-Gd**	**1-Y**
**Ln1-Zn1**	3.5718(6)	3.5408(4)
**Ln1-O1**	2.410(4)	2.372(3)
**Ln1-O2**	2.346(3)	2.316(2)
**Ln1-O3**	2.315(3)	2.268(2)
**Ln1-O4**	2.410(4)	2.370(2)
**Zn1-N1**	2.150(4)	2.155(3)
**Zn1-N2**	2.109(4)	2.109(3)
**Zn1-O2**	2.083(3)	2.072(2)
**Zn1-O3**	2.131(3)	2.133(2)
**Zn1-Cl1**	2.2089(14)	2.2113(9)
**Ln1···Ln1**	9.2275(5)	9.2189(4)
**Ln1···Cl1**	4.5992(14)	4.5668(9)
**Gd1···H1**	4.18022(17)	4.13323(11)
**Gd1···H2**	4.6075(4)	4.5992(4)
**Gd1···H3**	4.4995(3)	4.4737(2)
**Gd1···H4**	4.1700(3)	4.1386(3)
**$C_g \cdots C_g$ distance**	7.897(3)	7.859(2)
**Ln1-O2-Zn1**	107.35(14)	107.46(10)
**Ln1-O3-Zn1**	106.84(14)	107.10(10)
**Zn1-Ln1-Zn1**	109.59(2)	109.402(15)

Table S2 (continuation). Selected bond lengths (Å), interatomic distances (Å) and angles (º) for compounds **1-Gd** and **1-Y**.

	**1-Gd**	**1-Y**
**O1-Ln1-O2**	71.10(12)	72.03(8)
**O1-Ln1-O4**	78.88(13)	77.16(9)
**O2-Ln1-O3**	67.97(11)	68.28(8)
**O3-Ln1-O4**	72.38(12)	73.09(8)
**O2-Zn1-O3**	76.36(13)	75.46(9)
**O2-Zn1-Cl1**	107.28(10)	107.83(7)
**O3-Zn1-Cl1**	102.23(10)	102.09(7)
**Ring···ring angle**	152.19	151.63

Continuous Shape Measurements

Table S3. Continuous Shape Measurements (CShM) for LnO_8 in compounds **1-Gd** and **1-Y**.

**Label**	**Gd1**	**Y1**
**OP-8**	30.343	30.140
**HPY-8**	22.812	23.145
**HBPY-8**	16.210	16.733
**CU-8**	10.351	10.467
**SAPR-8**	0.617	0.509
**TDD-8**	2.985	2.785
**JGBF-8**	15.030	15.466
**JETBPY-8**	25.556	26.213
**JBTP-8**	3.392	3.308
**BTPR-8**	2.779	2.668
**JSD-8**	5.871	5.766
**TT-8**	11.200	11.216
**ETBPY-8**	21.279	21.852

Abbreviations: OP-8: Octagon (D_{8h}); HPY-8: Heptagonal pyramid (C_{7v}); HBPY-8: Hexagonal bipyramid (D_{6h}); CU-8: Cube (O_h); SAPR-8: Square antiprism (D_{4d}); TDD-8: Triangular dodecahedron (D_{2d}); JGBF-8: Johnson - Gyrobifastigium J26 (D_{2d}); JETBPY-8: Johnson – Elongated triangular bipyramid J14 (D_{3h}); JBTP-8: Johnson – Biaugmented trigonal prism J50 (C_{2v}); BTPR-8: Biaugmented trigonal prism (C_{2v}); JSD-8: Snub disphenoid J84 (D_{2d}); TT-8: Triakis tetrahedron (T_d); ETBPY-8: Elongated trigonal bipyramid (D_{3h}).

Table S4. Continuous Shape Measurements (CShM) for ZnO_2N_2Cl in compounds **1-Gd** and **1-Y**.

Label	Gd1	Y1
**PP-5**	32.402	32.522
**vOC-5**	3.399	3.531
**TBPY-5**	4.186	4.095
**SPY-5**	0.598	0.681
**JTBPY-5**	7.360	7.231

Abbreviations: PP-5: Pentagon (D_{5h}); vOC-5: Vacant octahedron - Johnson square pyramid J1 (C_{4v}); TBPY-5: Trigonal bipyramid (D_{3h}); SPY-5: Square pyramid (C_{4v}); JTBPY-5: Johnson trigonal bipyramid J12 (D_{3h}).

Powder X-Ray Diffraction

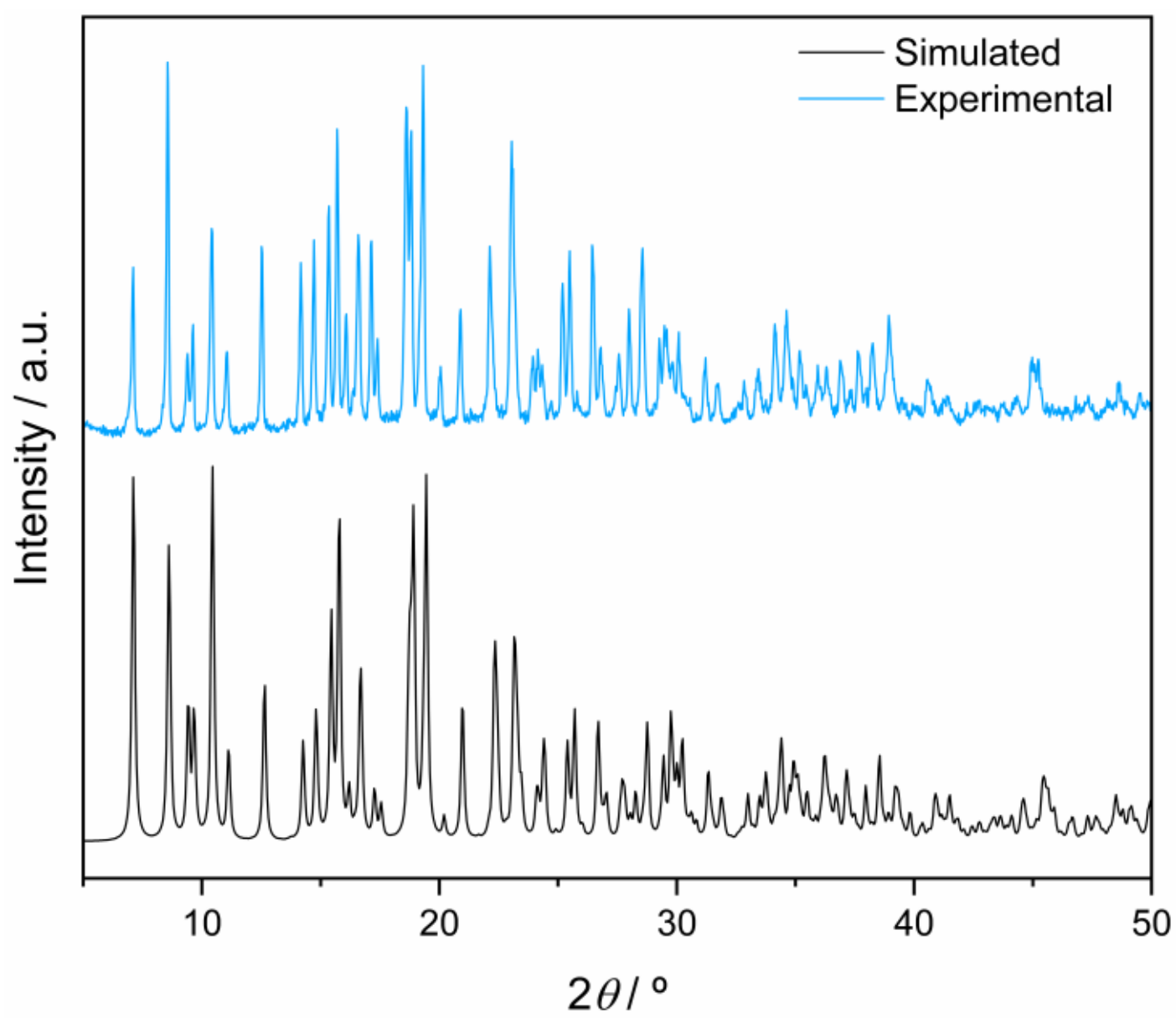

Figure S4. Comparison between the experimental powder X-ray diffraction pattern for the single crystals of **1-Gd** and the simulated pattern extracted from SCXRD data.

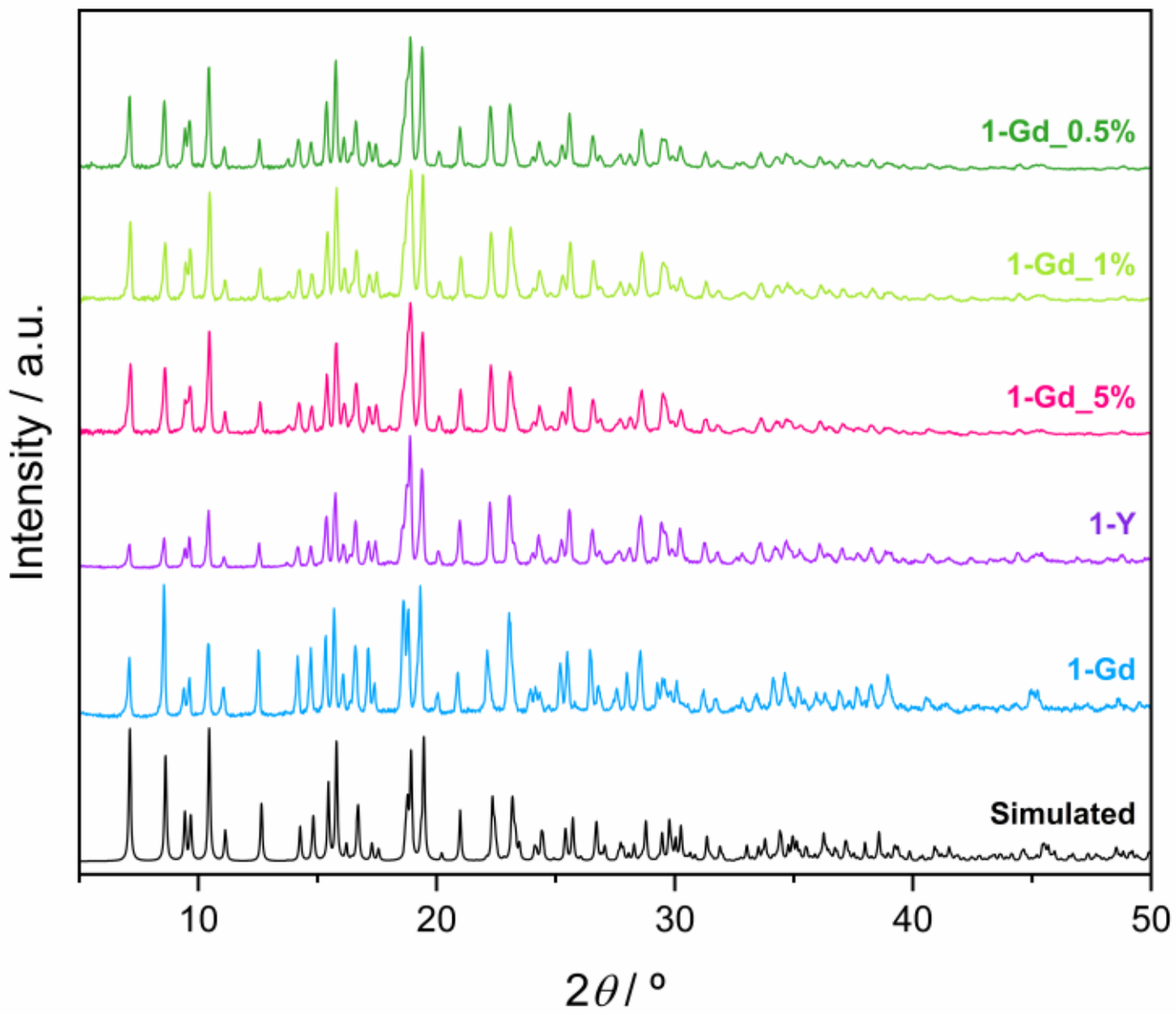

Figure S5. Comparison between the experimental powder X-ray diffraction pattern for the single crystals of **1-Gd**, **1-Y**, **1-Gd_5%**, **1-Gd_1%**, **1-Gd_0.5%** and the simulated pattern from SCXRD data.

CW-EPR Spectroscopy

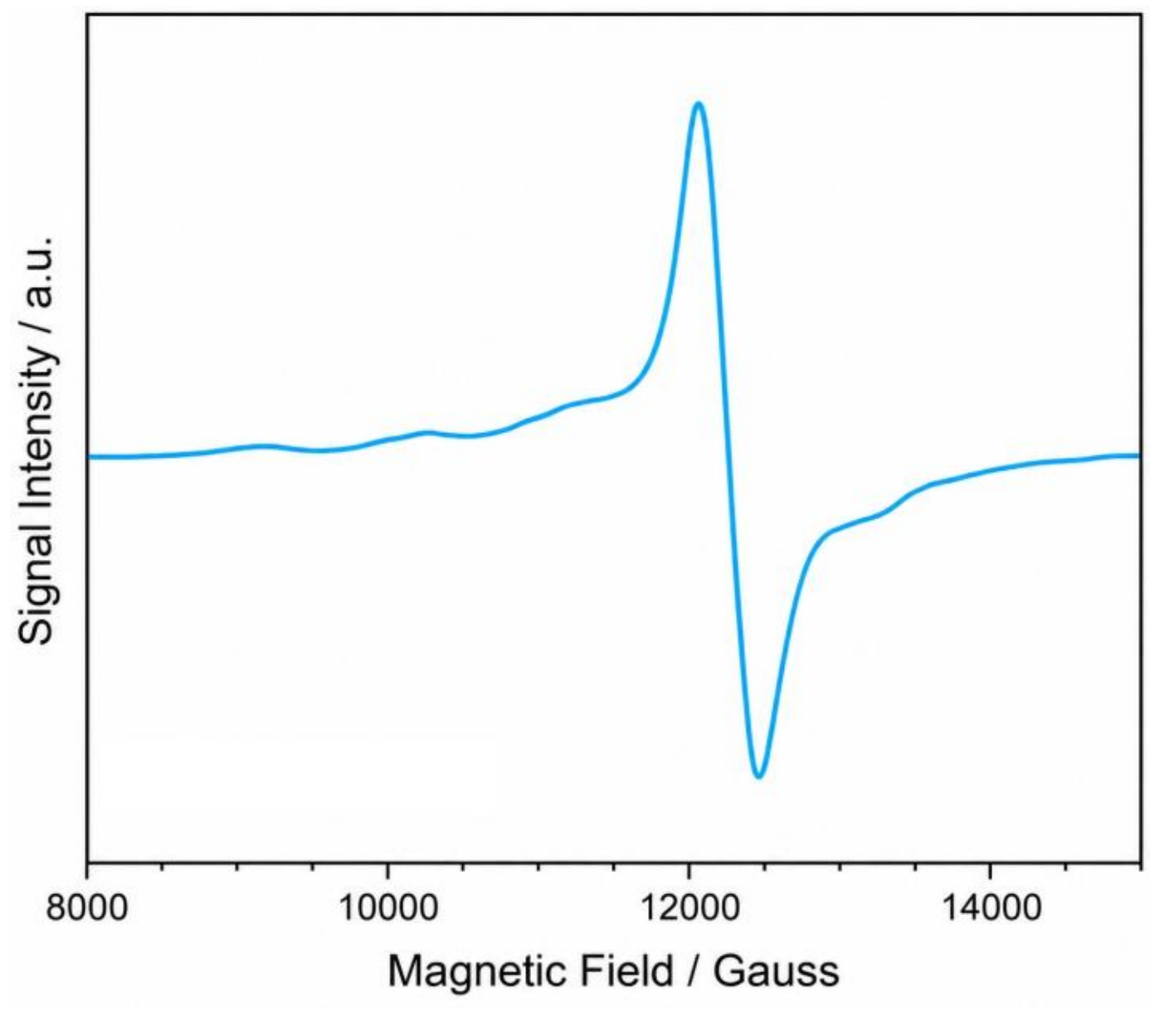

Figure S6. Q-band CW-EPR spectrum recorded for **1-Gd** measured at room temperature.

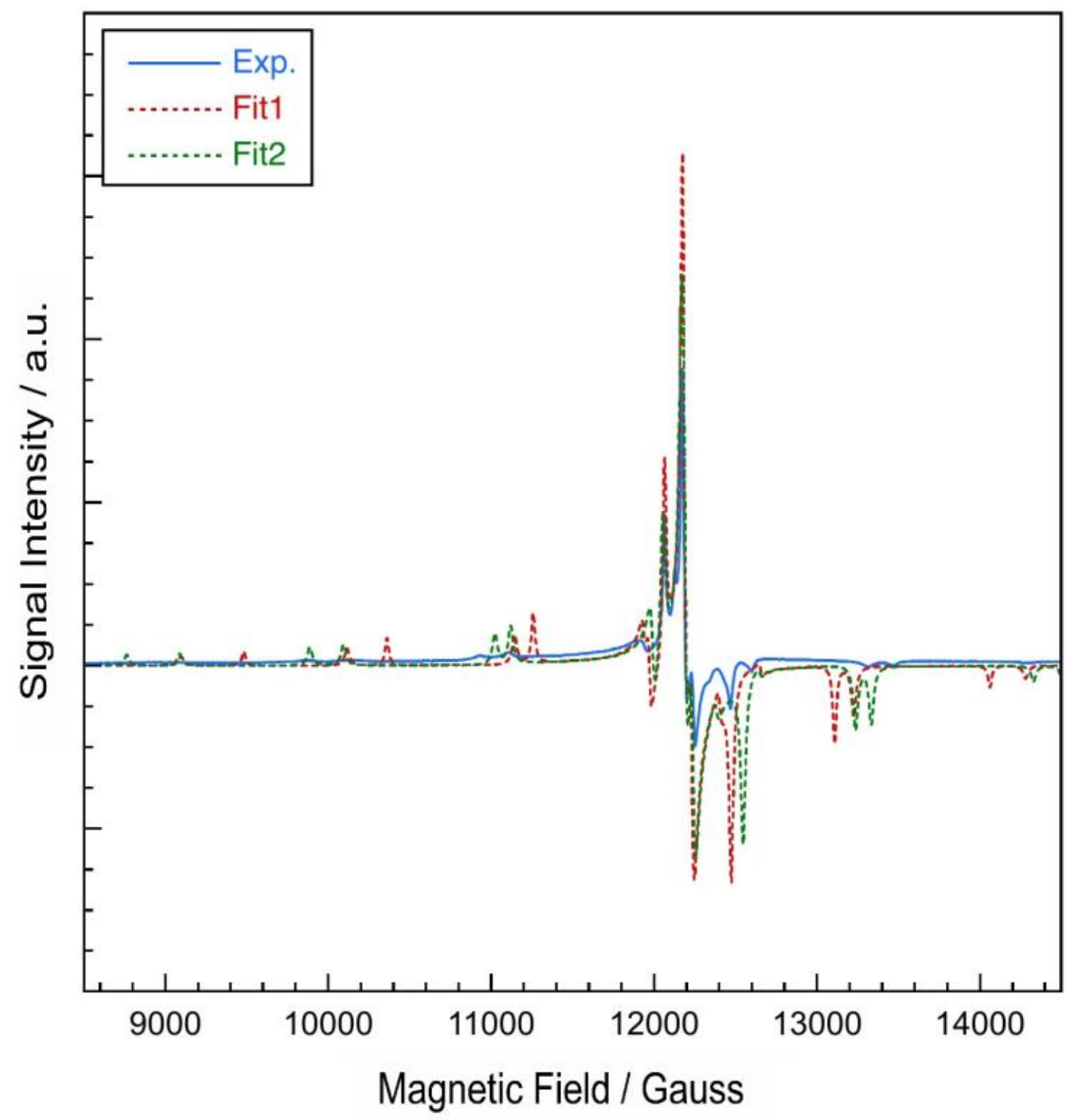

Figure S7. Q-band CW-EPR spectrum recorded for **1-Gd_5%** (solid blue line), together with its simulations (dashed red and green lines), measured at room temperature.

***Ab-initio* Calculations**

Table S5. Comparison between the *D* and *E* parameters and *g*-tensors determined from the best fitting to the experimental Q-band CW-EPR spectrum in **1-Gd_5%** and *ab-initio* calculations for the Gd^{III} ion in **1-Gd**.

	***D* / cm^{-1}**	***E* / cm^{-1}**	g_x	g_y	g_z
**1-Gd_5%$_{exp}$ (Fit 1)**	0.0484	0.0125	1.994	1.990	1.993
**1-Gd_5%$_{exp}$ (Fit 2)**	0.0537	0.0149	1.990	1.991	1.993
**1-Gd$_{theor}$**	0.0262	-0.0065	1.994	1.994	1.994

Table S6. *Ab-initio* computed crystal field parameters for the Gd ions in **1-Gd**.

k	q	B_k^q / cm^{-1}
2	-2	0.63E-01
	-1	-0.96E-07
	0	-0.11E-02
	1	0.22E-06
	2	0.41E-04
4	-4	-0.23E-04
	-3	0.84E-09
	-2	0.41E-05
	-1	0.18E-08
	0	-0.80E-06
	1	0.33E-08
	2	0.14E-04
	3	-0.74E-08
	4	-0.41E-04
6	-6	-0.54E-07
	-5	-0.89E-09
	-4	-0.19E-07
	-3	0.17E-09
	-2	0.57E-08
	-1	-0.69E-10
	0	0.82E-09
	1	-0.29E-09
	2	0.89E-08
	3	-0.10E-09
	4	0.12E-07
	5	-0.24E-10
	6	0.52E-08

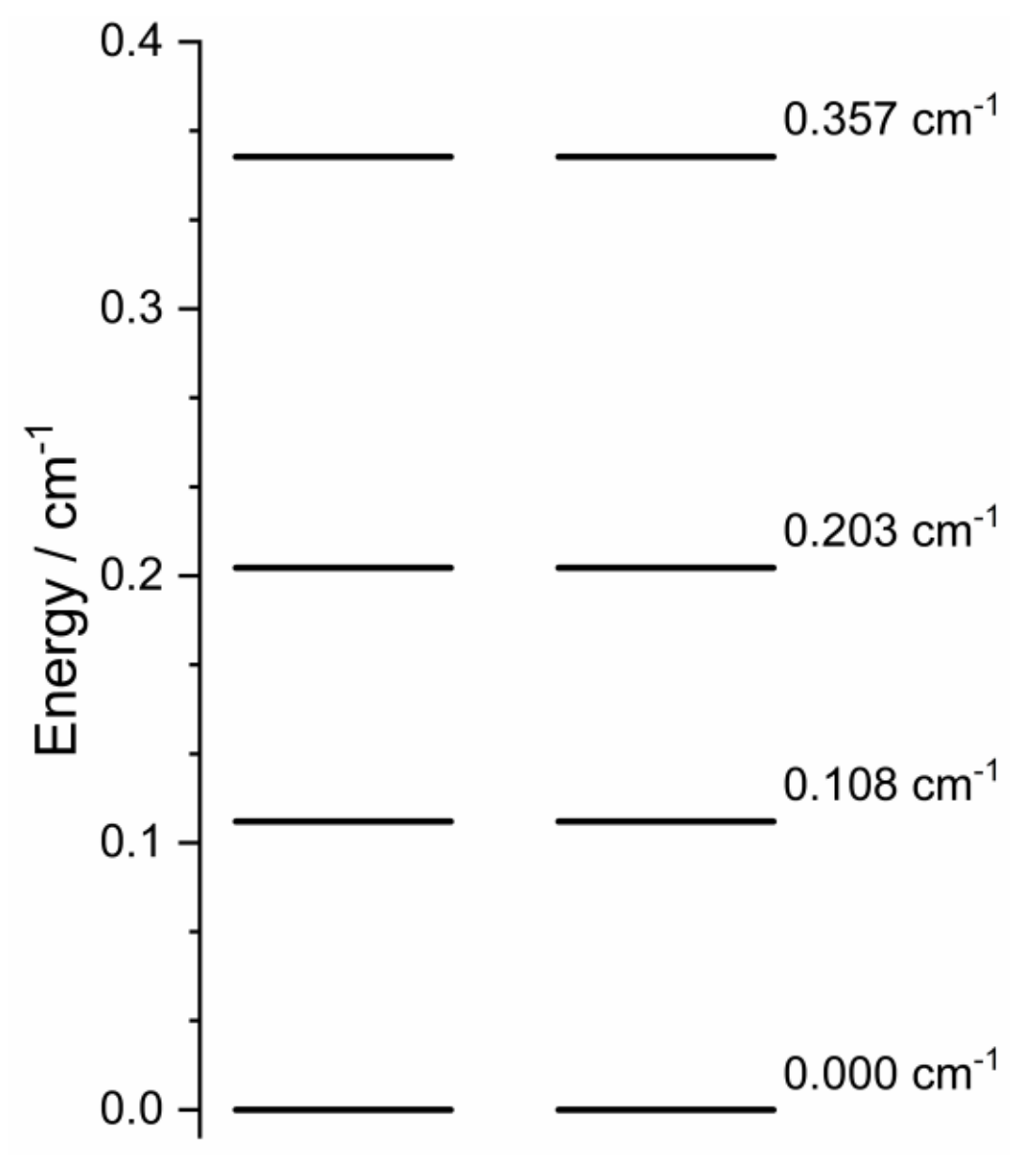

Figure S8. *Ab-initio* computed energies diagram of low-lying doublets of Gd^{III} ions in **1-Gd**.

Magnetic properties

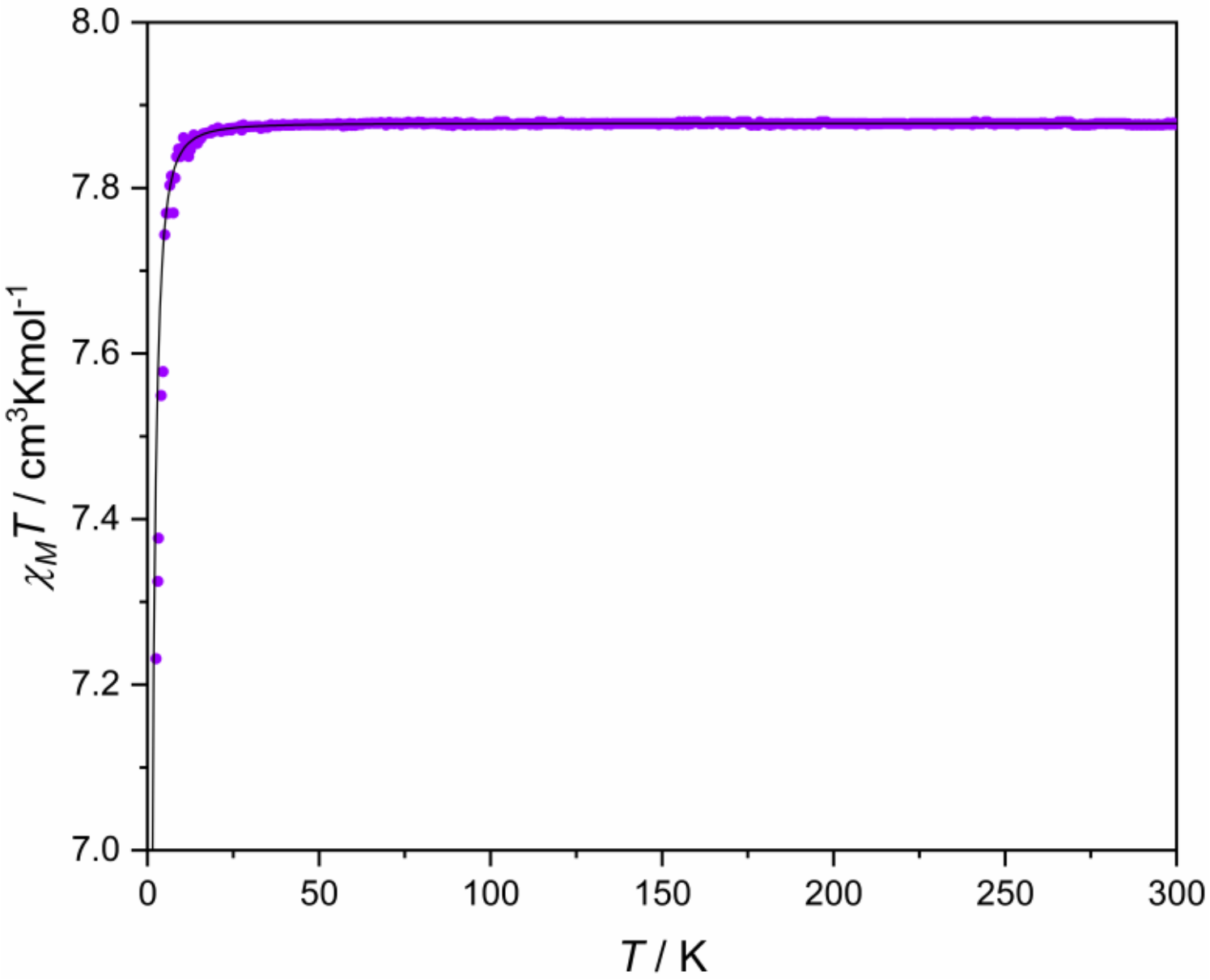

Figure S9. Temperature dependence of the $\chi_M T$ product at 1000 Oe for **1-Gd**. The solid black line represents the calculated curve with the zero-field splitting parameters deduced from the CW-EPR spectrum.

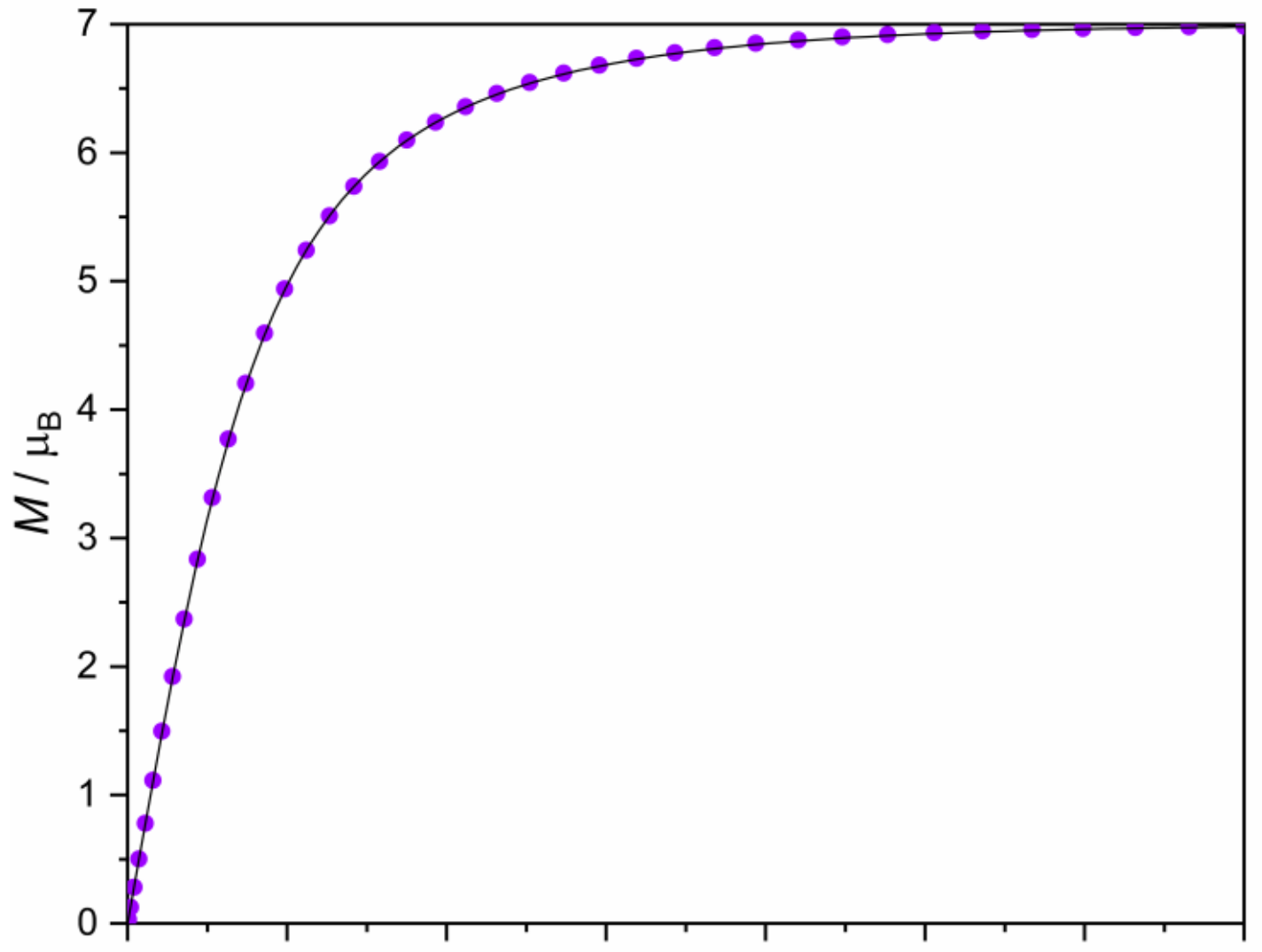

Figure S10. Field dependent magnetization curve measured at 2 K for **1-Gd**. The solid black line represents the calculated curve with the zero-field splitting parameters deduced from the CW-EPR spectrum.

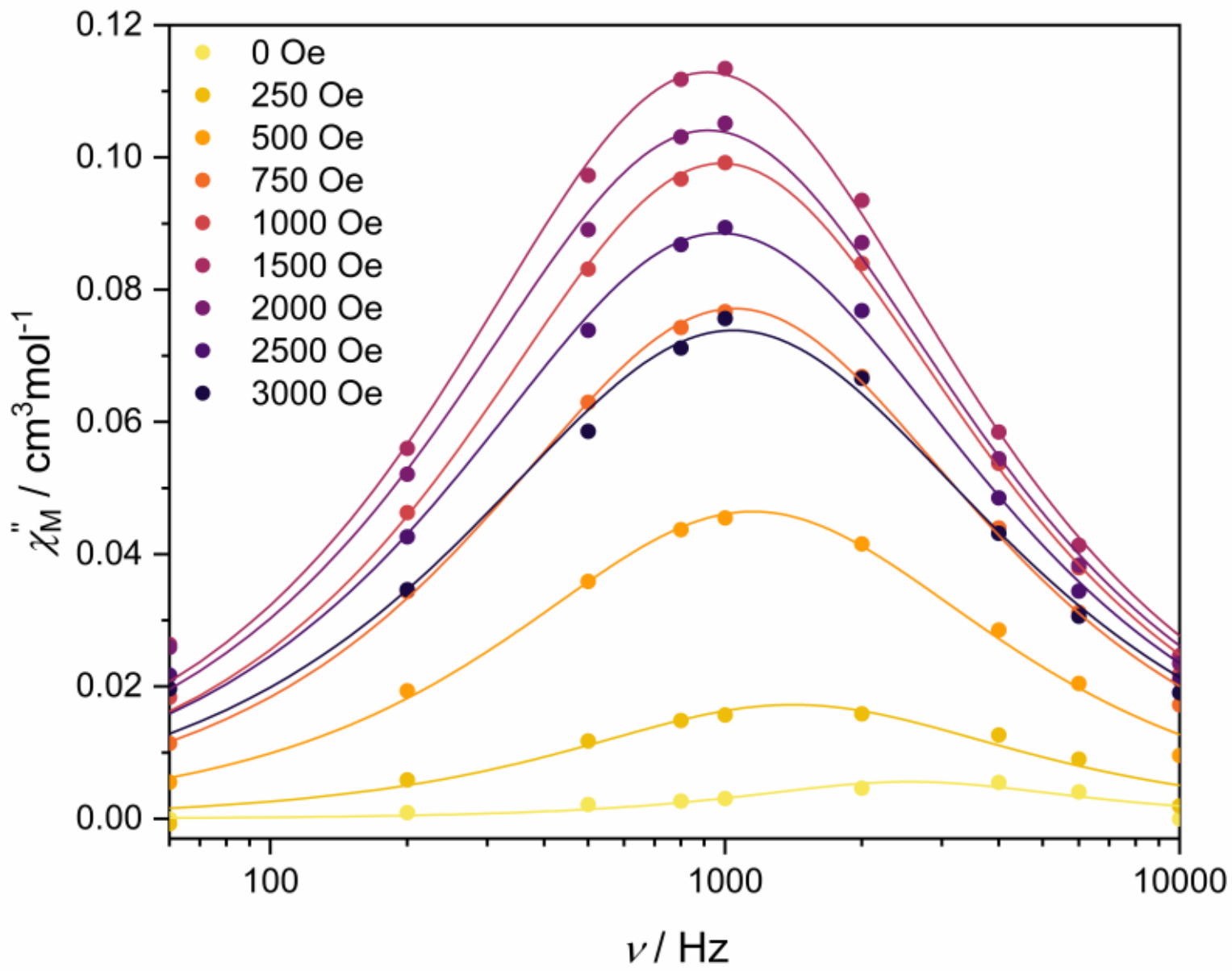

Figure S11. Frequency dependence of the out-of-phase (χ''_M) component of the *ac* susceptibilities at different *dc* fields and 2 K for compound **1-Gd**. The solid lines represent the best fit of the data.

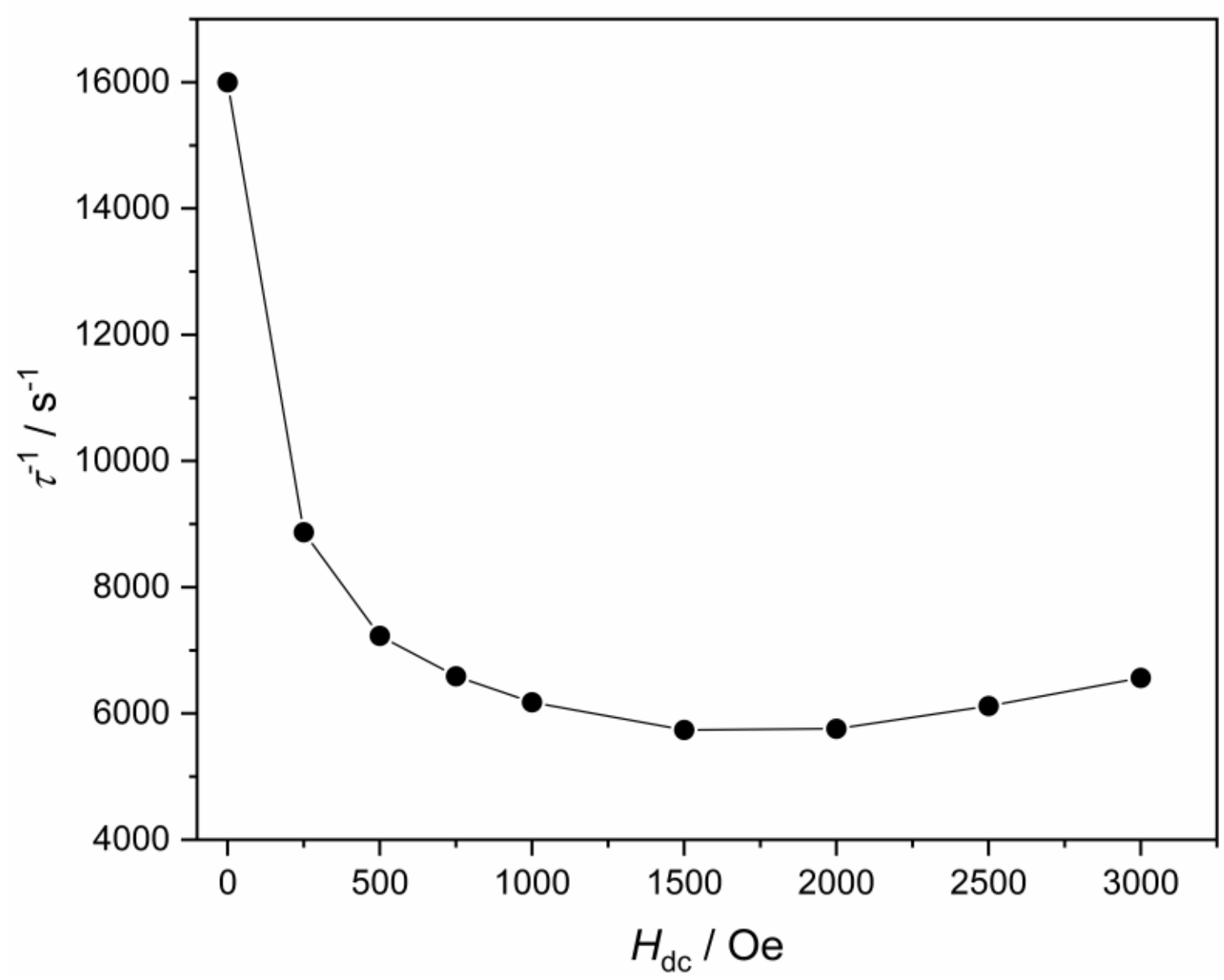

Figure S12. Field dependence of the relaxation times for **1-Gd** at 2 K. The solid line represents a guide to the eye.

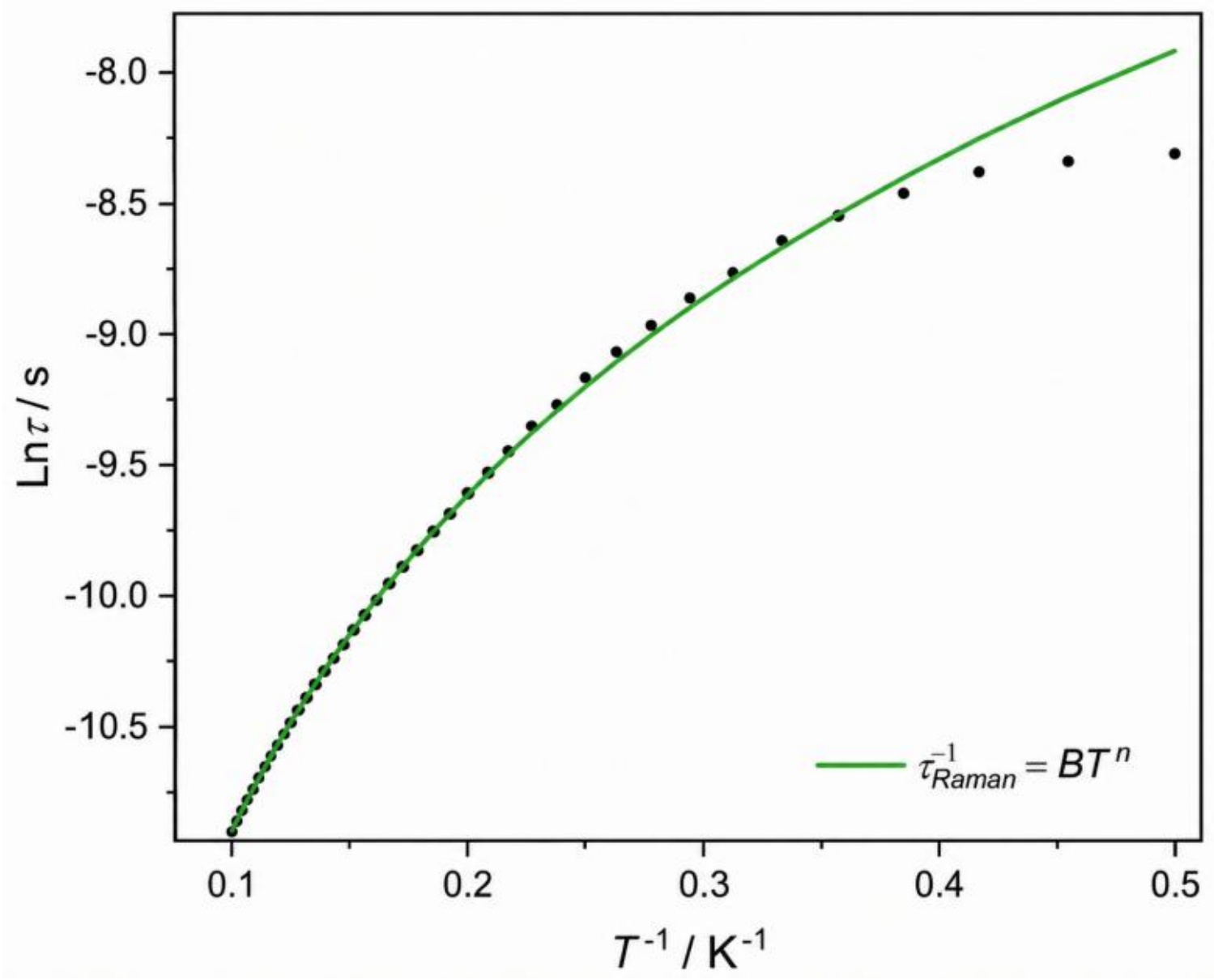

Figure S13. The best fitting of the experimental data to Eq. 2 as described in the main text (green line). The experimental data are denoted by circles.

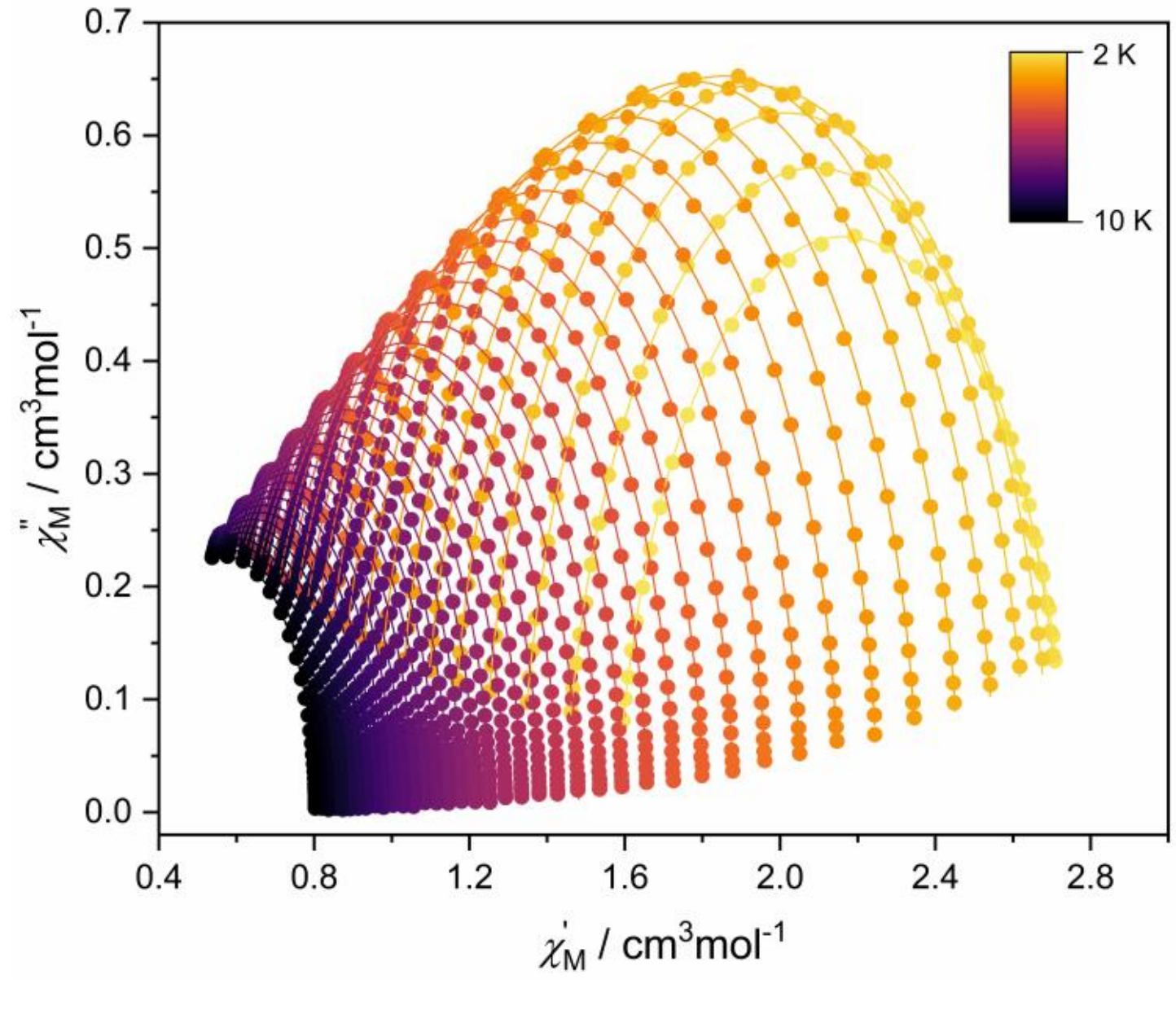

Figure S14. Cole-Cole plot for **1-Gd** at H_{dc} = 1500 Oe. The experimental data are denoted by circles; solid lines represent the best fit to the generalized Debye model.

Table S7. Best fitting parameters of *ac* magnetic data according to the generalized Debye model for **1-Gd**.

T / K	χ_S / cm^{3}mol^{-1}	χ_T / cm^{3}mol^{-1}	α	τ / s
**2**	1.5794(8)	2.7473(11)	0.085(2)	2.47E-04(2)
**2.2**	1.4443(5)	2.7357(5)	0.0772(13)	2.393E-04(12)
**2.4**	1.3318(8)	2.6991(10)	0.062(2)	2.296E-04(8)
**2.6**	1.2337(6)	2.6381(7)	0.0542(13)	2.120E-04(7)
**2.8**	1.1497(8)	2.5568(7)	0.047(2)	1.940E-04(4)
**3**	1.0778(5)	2.4613(4)	0.0397(10)	1.759E-04(3)
**3.2**	1.0097(10)	2.3556(6)	0.040(2)	1.559E-04(5)
**3.4**	0.9541(11)	2.2524(6)	0.033(2)	1.416E-04(3)
**3.6**	0.9010(8)	2.1531(4)	0.0334(13)	1.275E-04(2)
**3.8**	0.8558(6)	2.0556(2)	0.0293(10)	1.151E-04(2)
**4**	0.8144(9)	1.9644(3)	0.0265(14)	1.044E-04(2)
**4.2**	0.7732(14)	1.8811(4)	0.032(2)	9.41E-05(2)
**4.4**	0.739(2)	1.802(3)	0.031(2)	8.68E-05(3)
**4.6**	0.708(2)	1.7271(3)	0.027(2)	7.89E-05(2)
**4.8**	0.680(2)	1.6586(3)	0.025(2)	7.280E-05(10)
**5**	0.6526(8)	1.59384(11)	0.0251(10)	6.731E-05(13)
**5.2**	0.626(2)	1.5370(2)	0.030(2)	6.228E-05(12)
**5.4**	0.6056(11)	1.48034(11)	0.0253(12)	5.808E-05(9)
**5.6**	0.5861(15)	1.42817(14)	0.020(2)	5.413E-05(9)
**5.8**	0.5640(14)	1.38064(12)	0.024(2)	5.075E-05(8)
**6**	0.5484(8)	1.335077(6)	0.0193(8)	4.761E-05(7)
**6.2**	0.5290(9)	1.29331(6)	0.0227(10)	4.476E-05(9)
**6.4**	0.512(2)	1.25299(11)	0.023(2)	4.222E-05(9)
**6.6**	0.499(2)	1.21584(11)	0.021(2)	3.989E-05(10)
**6.8**	0.481(2)	1.18059(12)	0.026(2)	3.777E-05(10)
**7**	0.468(2)	1.14689(8)	0.025(2)	3.586E-05(10)
**7.2**	0.4584(14)	1.11510(6)	0.0200(13)	3.409E-05(6)
**7.4**	0.442(2)	1.08551(7)	0.026(2)	3.241E-05(6)
**7.6**	0.432(3)	1.05644(10)	0.023(3)	3.085E-05(9)
**7.8**	0.417(3)	1.03034(10)	0.030(3)	2.948E-05(8)
**8**	0.418(2)	1.00326(7)	0.013(2)	2.812E-05(4)
**8.2**	0.406(3)	0.97941(7)	0.017(2)	2.690E-05(9)
**8.4**	0.386(3)	0.95775(6)	0.032(2)	2.572E-05(6)
**8.6**	0.388(3)	0.93346(7)	0.017(3)	2.468E-05(8)
**8.8**	0.374(2)	0.91290(4)	0.023(2)	2.372E-05(12)
**9**	0.3688(7)	0.892589(13)	0.0197(5)	2.272E-05(11)
**9.2**	0.363(2)	0.87333(3)	0.0183(12)	2.176E-05(12)
**9.4**	0.346(2)	0.85570(4)	0.0300(14)	2.090E-05(8)
**9.6**	0.343(4)	0.83734(7)	0.025(3)	2.005E-05(11)
**9.8**	0.334(4)	0.82002(8)	0.027(3)	1.927E-05(6)
**10**	0.340(4)	0.80314(6)	0.013(3)	1.847E-05(4)

Pulsed EPR Spectroscopy

The echo-detected field swept EPR spectra were recorded using a two-pulse Hahn-echo sequence (π/2-τ-π-τ-echo) under variation of the static B_0 magnetic field. The pulse parameters were selected to optimize the echo intensity. Thus, π pulse length of 32 ns and τ = 200 ns were chosen for all the diluted samples.

The echo decay curves were collected by application of the above-mentioned sequence at the selected B_0 field position given in the main text and variable temperatures with varying τ (starting from τ = 200 ns). The duration of π/2 and π pulses were set to be 16 ns and 32 ns, respectively. Phase memory times, T_m, were calculated by fitting the integrals of the Hahn-echo decay to a monoexponential model as described in the main text.

Spin-lattice relaxation times T_1 were obtained at the main resonance, as for the phase memory times, using an inversion recovery sequence (π-t-π/2-τ-π-τ-echo) with variable t and fixed τ chosen to be 200 ns for a π/2 pulse of 16 ns. The experimental data were fitted to a biexponential model as described in the main text.

Transient nutation experiments were performed using a variable-length nutation pulse (tp) to rotate the magnetization through a tipped angle defined as $\theta = g\mu_B B_1 t_p/\hbar$, within the standard sequence (tp-t-π/2-τ-π-τ-echo) at different attenuations of the microwave power.

In these experiments, the length of the tp pulse was gradually incremented in 4 ns, whereas those of the π/2 and π pulses were kept fixed and optimized for each microwave power in order to maximize the echo intensity. Rabi frequencies, Ω_R, were obtained by zero-filling the Rabi oscillation curves followed by a fast Fourier transform (FTT).

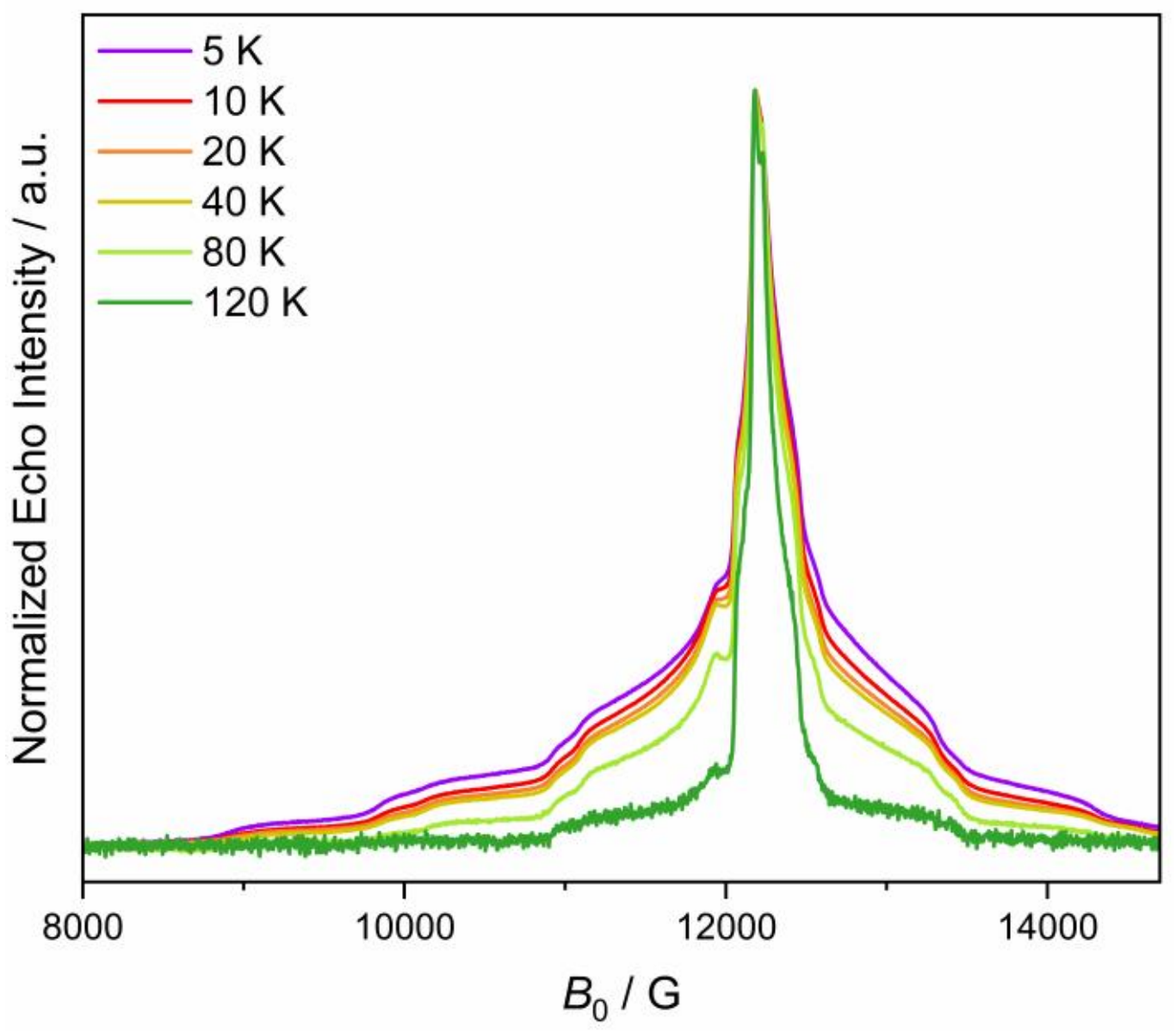

Figure S15. Echo-detected field-swept EPR spectra (34 GHz; π/2 pulse of 16 ns) of **1-Gd_1%** at different temperatures.

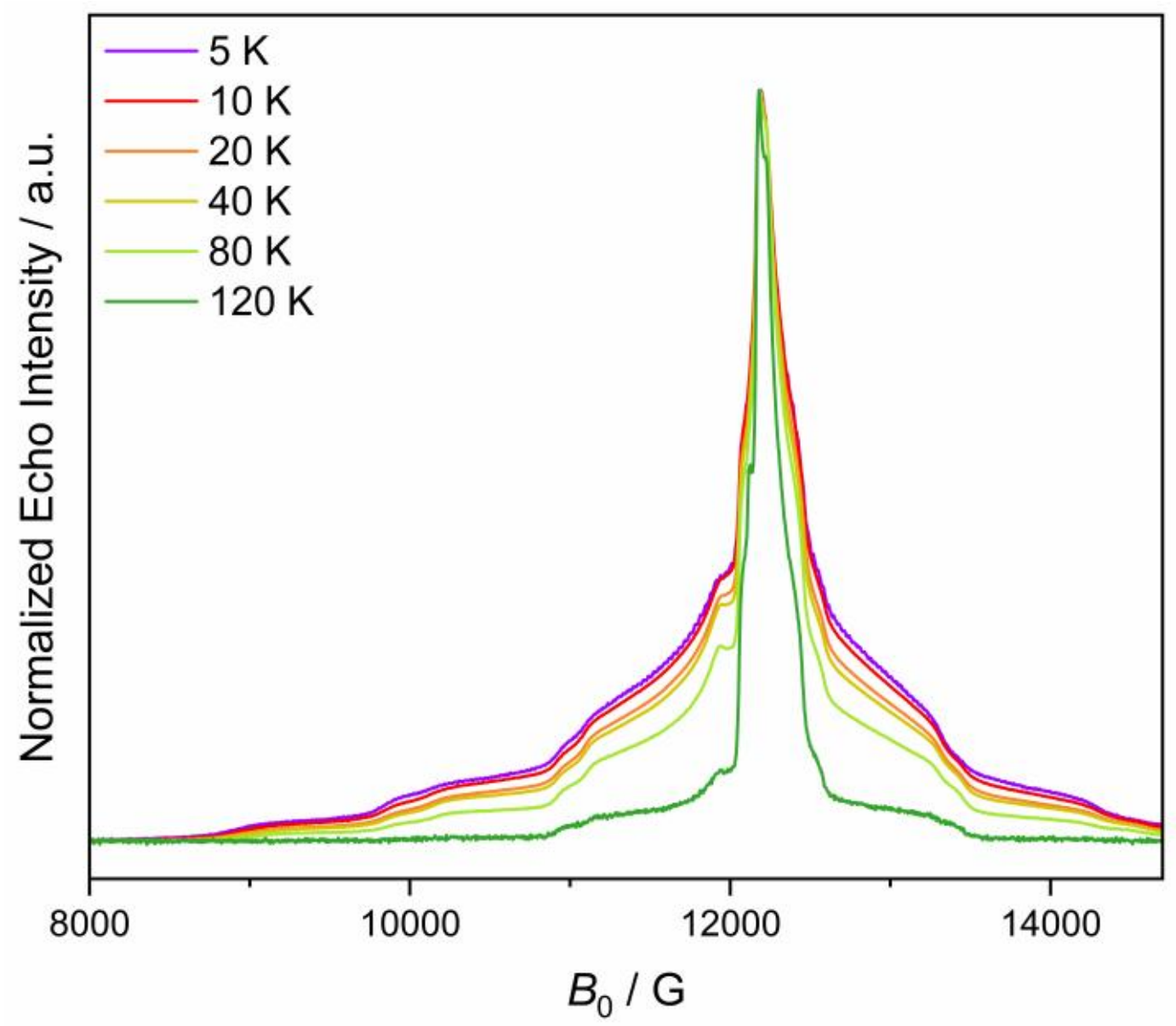

Figure S16. Echo-detected field-swept EPR spectra (34 GHz; π/2 pulse of 16 ns) of **1-Gd_0.5%** at different temperatures.

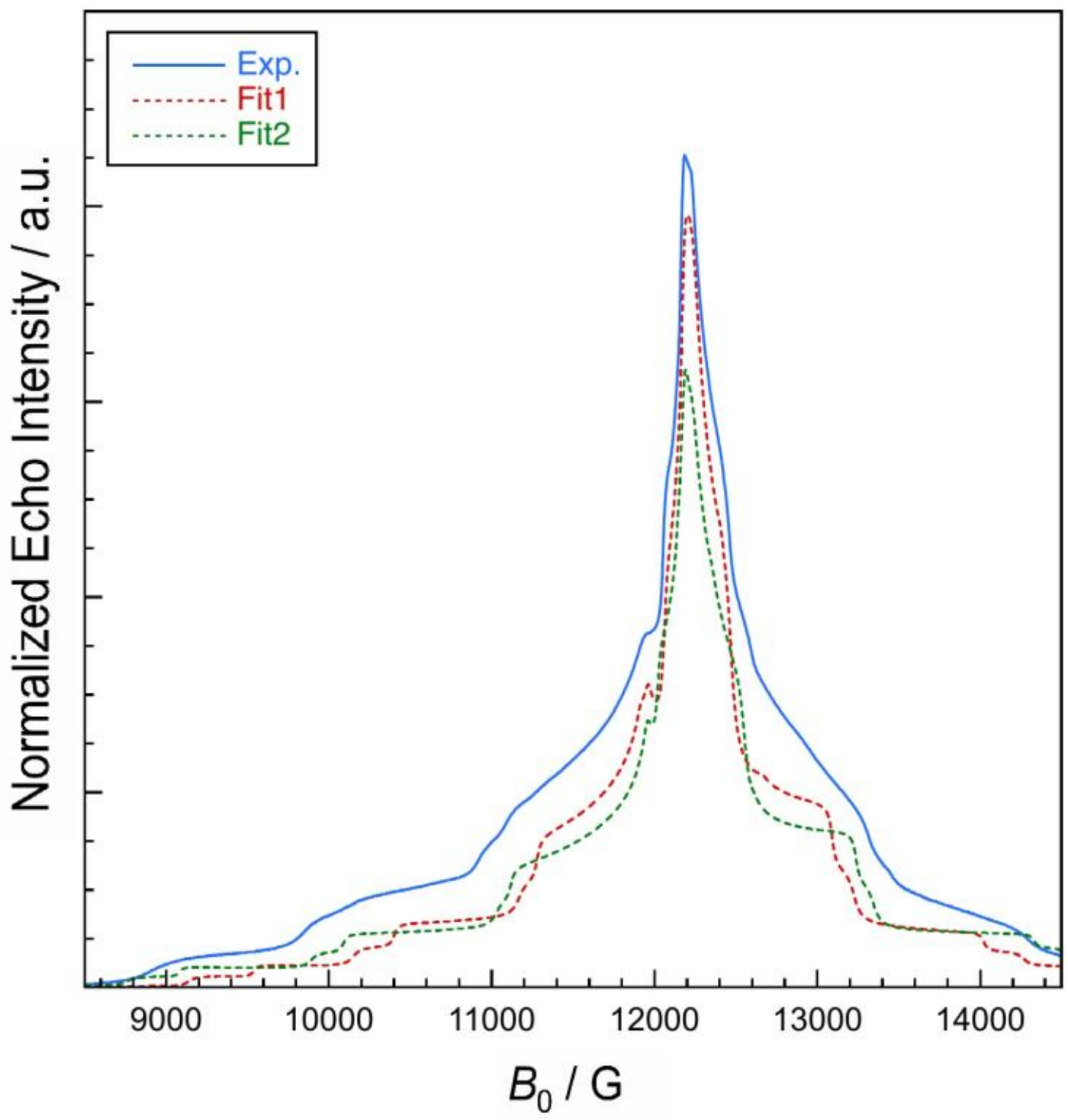

Figure S17. Echo-detected field-swept EPR spectra (34 GHz; $\pi/2$ pulse of 16 ns) recorded for **1-Gd_5%** at 5 K (solid blue line), together with its simulations as described in the main text (dashed red and green lines).

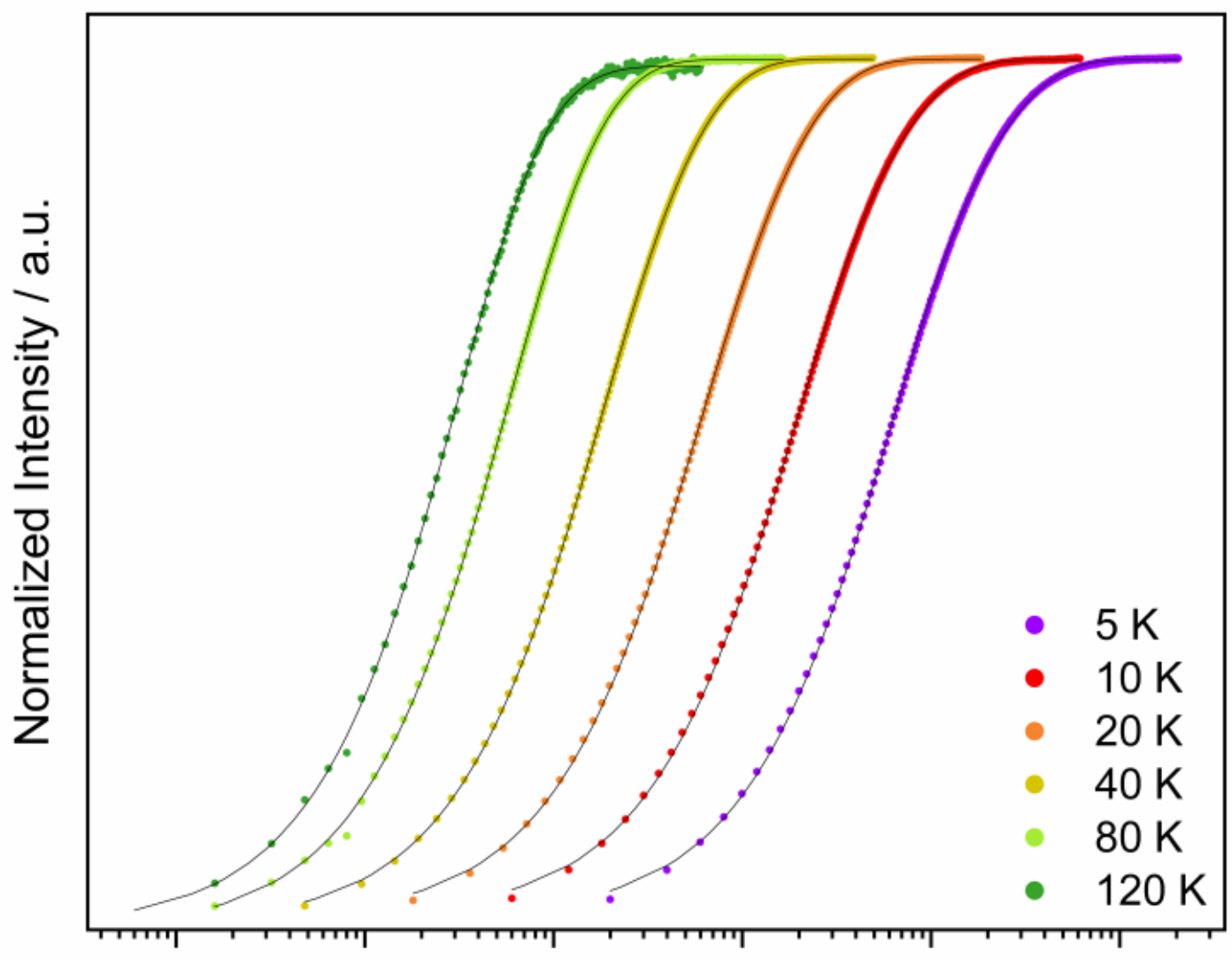

Figure S18. Normalized echo intensities as a function of time, t, in a standard inversion recovery sequence (34 GHz; $\pi/2$ pulse of 16 ns) for **1-Gd_5%** at B_0 = 12184 G and different temperatures. The solid lines are best fits to the biexponential model (Eq. 3) as described in the main text.

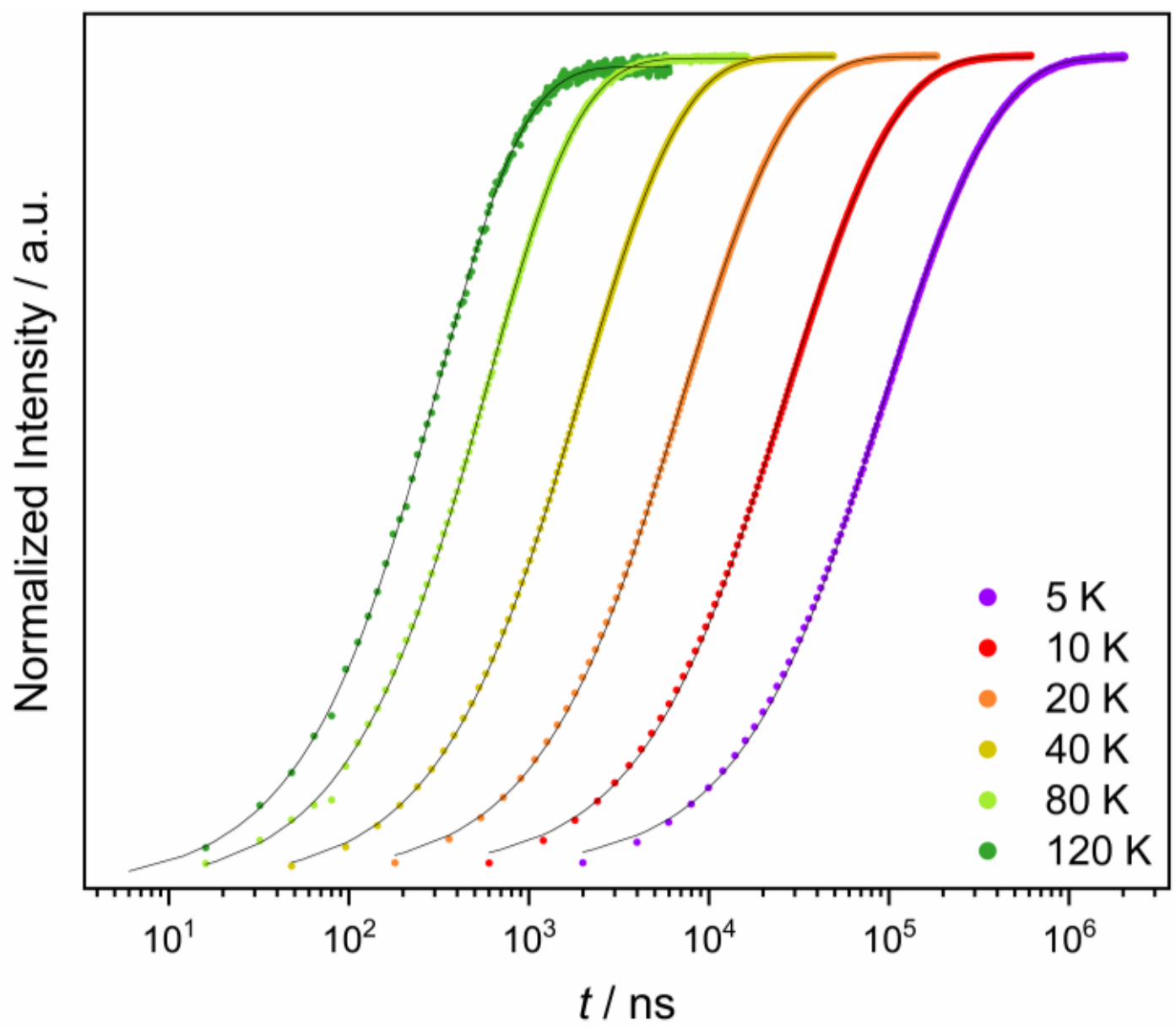

Figure S19. Normalized echo intensities as a function of time, *t*, in a standard inversion recovery sequence (34 GHz; $\pi/2$ pulse of 16 ns) for **1-Gd_1%** at B_0 = 12184 G and different temperatures. The solid lines are best fits to the biexponential model (Eq. 3) as described in the main text.

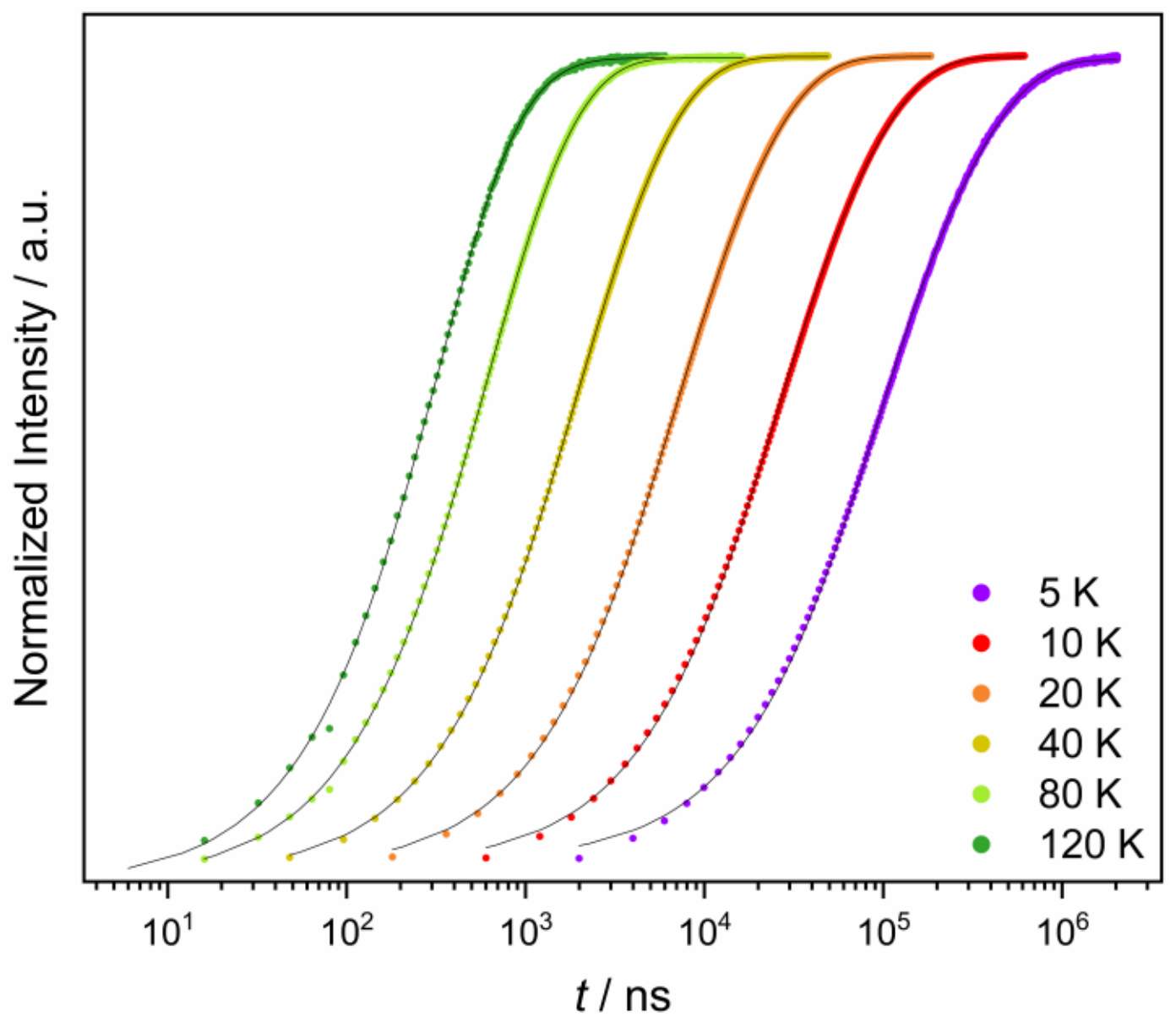

Figure S20. Normalized echo intensities as a function of time, *t*, in a standard inversion recovery sequence (34 GHz; $\pi/2$ pulse of 16 ns) for **1-Gd_0.5%** at B_0 = 12184 G and different temperatures. The solid lines are best fits to the biexponential model (Eq. 3) as described in the main text.

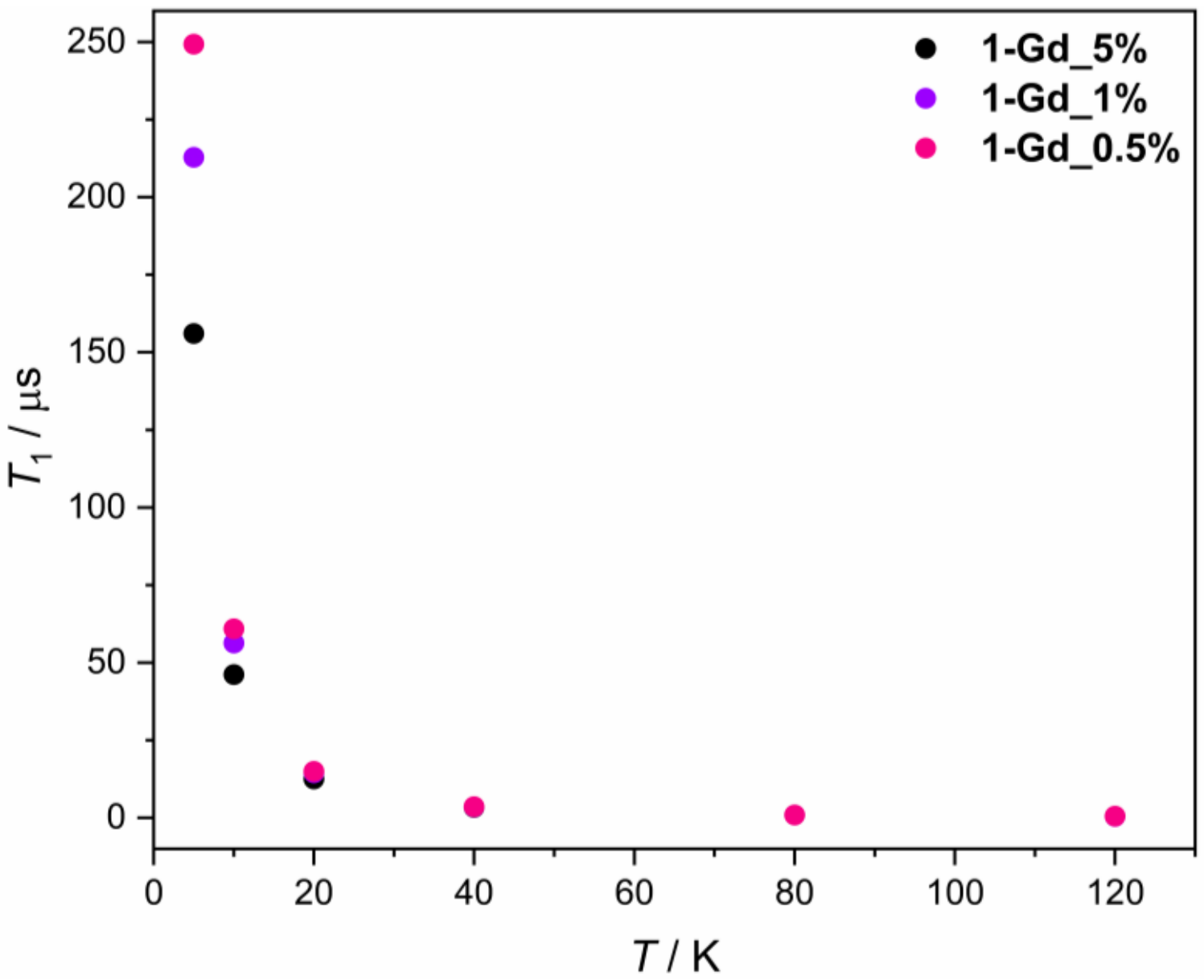

Figure S21. Temperature dependence of T_1 values of **1-Gd_5%**, **1-Gd_1%** and **1-Gd_0.5%**, at B_0 = 12184 G.

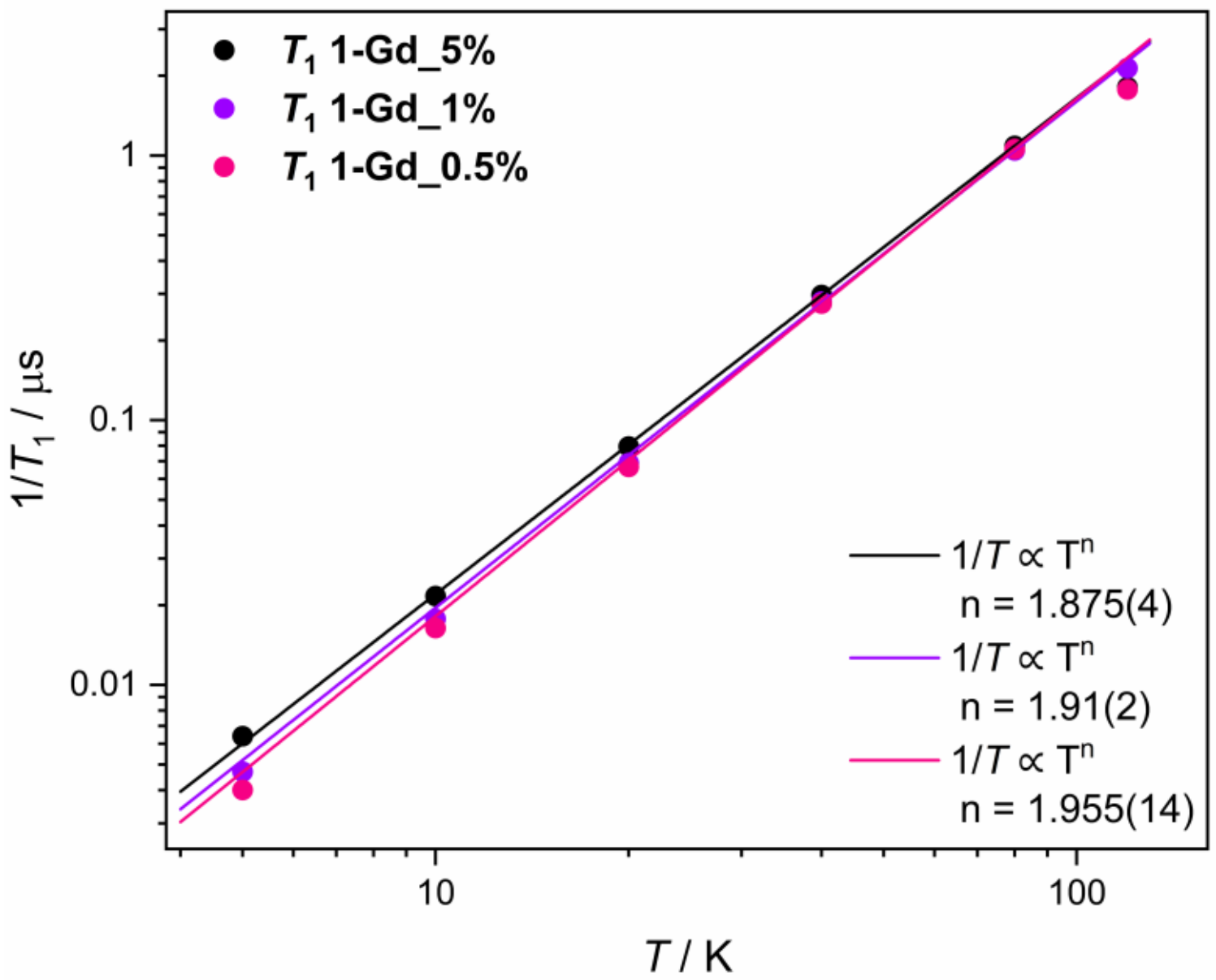

Figure S22. Inverse of the spin-lattice relaxation time, $1/T_1$, recorded at B_0 = 12184 G as a function of the temperature for samples **1-Gd_5%**, **1-Gd_1%** and **1-Gd_0.5%**. Solid lines represent the best fit of the data (excluding the last points as explained in the main text) to the power law $1/T_1 = AT^n$.

Table S8. Fitting parameters for T_1 for **1-Gd_5%** at different temperatures.

***T* / K**	**T_1 / µs**	**$T_{1,s}$ / µs**
**5**	156.1(7)	46.9(2)
**10**	46.2(2)	14.49(8)
**20**	12.57(5)	4.16(2)
**40**	3.363(10)	1.241(6)
**80**	0.919(5)	0.405(4)
**120**	0.55(3)	0.274(8)

Table S9. Fitting parameters for T_1 for **1-Gd_1%** at different temperatures.

***T* / K**	***T*₁ / µs**	***T*₁,s / µs**
**5**	212.8(9)	61.2(4)
**10**	56.3(2)	16.73(10)
**20**	14.52(5)	4.56(3)
**40**	3.56(1)	1.268(6)
**80**	0.956(7)	0.420(5)
**120**	0.47(3)	0.25(2)

Table S10. Fitting parameters for T_1 for **1-Gd_0.5%** at different temperatures.

***T* / K**	***T*₁ / µs**	***T*₁,s / µs**
**5**	249.3(12)	67.3(4)
**10**	60.9(2)	17.9(1)
**20**	15.01(5)	4.65(3)
**40**	3.627(9)	1.282(6)
**80**	0.944(4)	0.411(3)
**120**	0.56(2)	0.280(6)

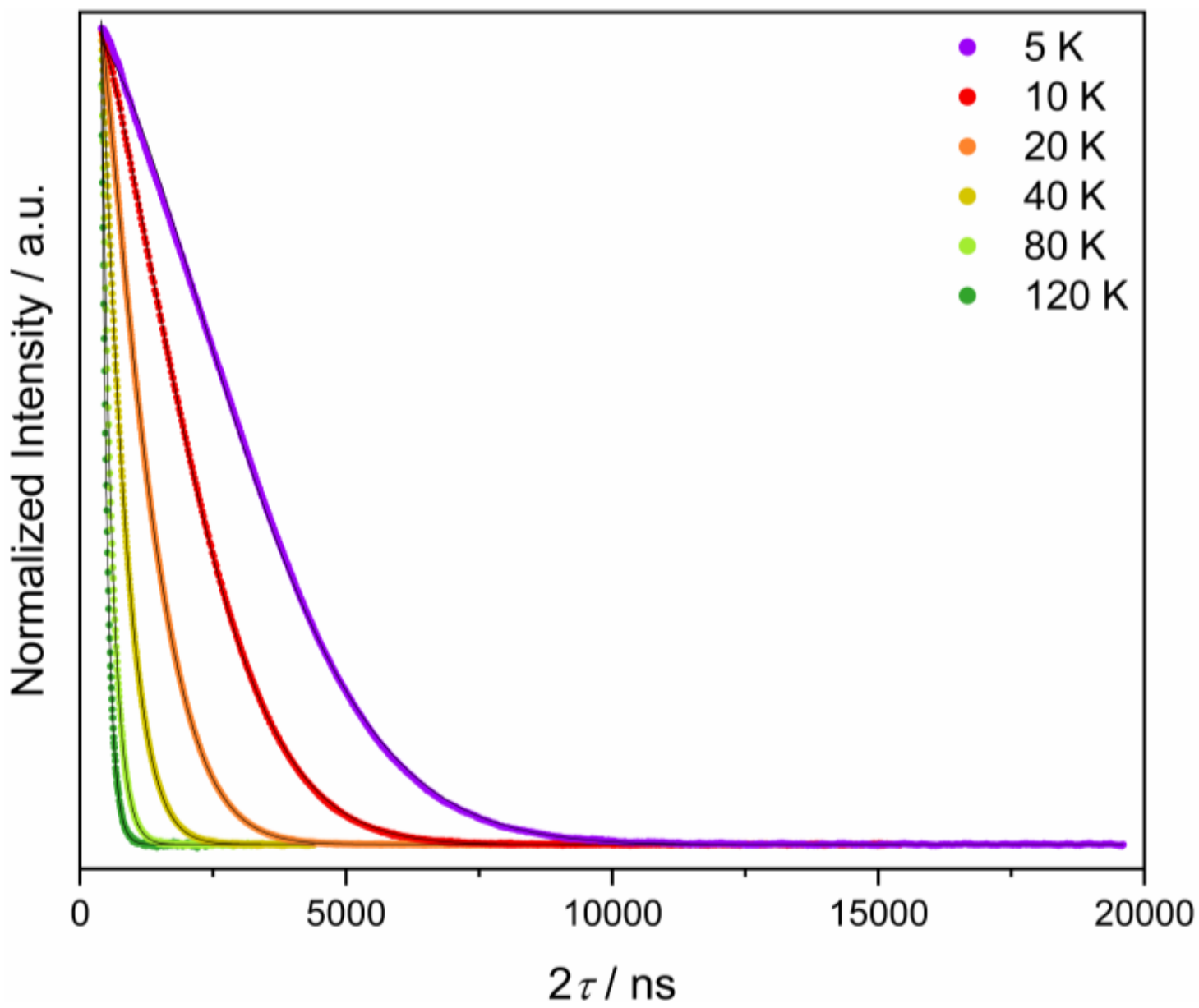

Figure S23. Normalized Hahn echo signals intensities as a function of their inter-pulse delay, 2τ, in a two-pulse Hahn-echo sequence (34 GHz; π/2 pulse of 16 ns) for **1-Gd_5%** at B_0 = 12184 G and different temperatures. The solid lines are best fits to the stretched exponential decay (Eq. 4) as described in the main text.

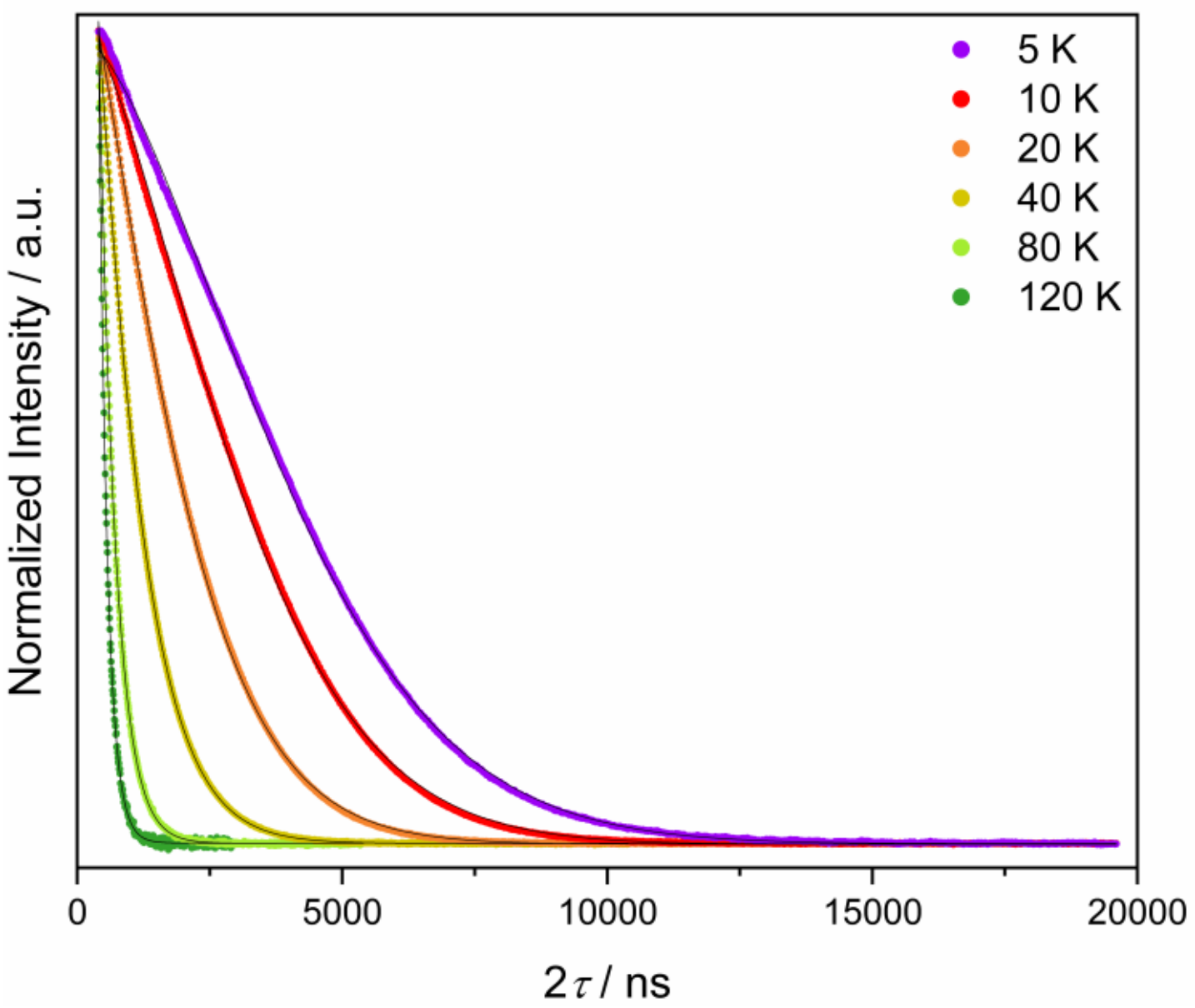

Figure S24. Normalized Hahn echo signals intensities as a function of their inter-pulse delay, 2τ, in a two-pulse Hahn-echo sequence (34 GHz; π/2 pulse of 16 ns) for **1-Gd_1%** at B_0 = 12184 G and different temperatures. The solid lines are best fits to the stretched exponential decay (Eq. 4) as described in the main text.

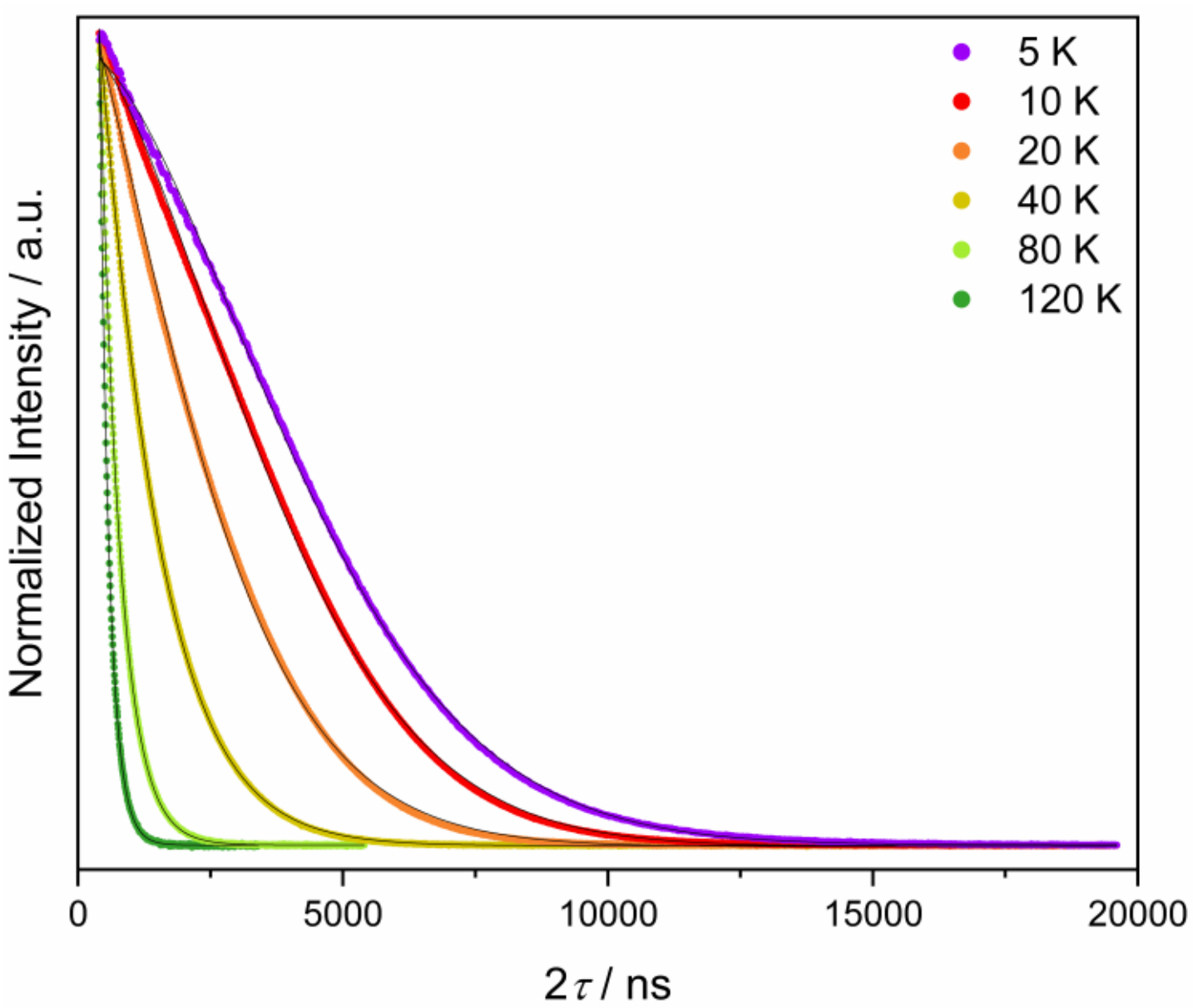

Figure S25. Normalized Hahn echo signals intensities as a function of their inter-pulse delay, 2τ, in a two-pulse Hahn-echo sequence (34 GHz; π/2 pulse of 16 ns) for **1-Gd_0.5%** at B_0 = 12184 G and different temperatures. The solid lines are best fits to the stretched exponential decay (Eq. 4) as described in the main text.

Table S11. Fitting parameters for T_m for **1-Gd_5%**.

***T* / K**	**T_m / µs**	***β***
**5**	3.706(3)	1.719(4)
**10**	2.3608(14)	1.610(2)
**20**	1.2684(5)	1.4728(8)
**40**	0.6536(9)	1.399(2)
**80**	0.2774(11)	1.216(3)
**120**	0.128(3)	1.040(10)

Table S12. Fitting parameters for T_m for **1-Gd_1%**.

***T* / K**	**T_m / µs**	***β***
**5**	4.505(5)	1.628(4)
**10**	3.425(4)	1.534(4)
**20**	2.077(2)	1.333(2)
**40**	0.9689(11)	1.1631(13)
**80**	0.339(2)	1.046(3)
**120**	0.137(5)	0.927(15)

Table S13. Fitting parameters for T_m for **1-Gd_0.5%**.

***T* / K**	**T_m / µs**	***β***
**5**	4.938(6)	1.681(5)
**10**	4.191(6)	1.634(5)
**20**	2.764(5)	1.365(4)
**40**	1.2156(13)	1.0898(11)
**80**	0.3832(11)	0.996(2)
**120**	0.147(2)	0.904(5)

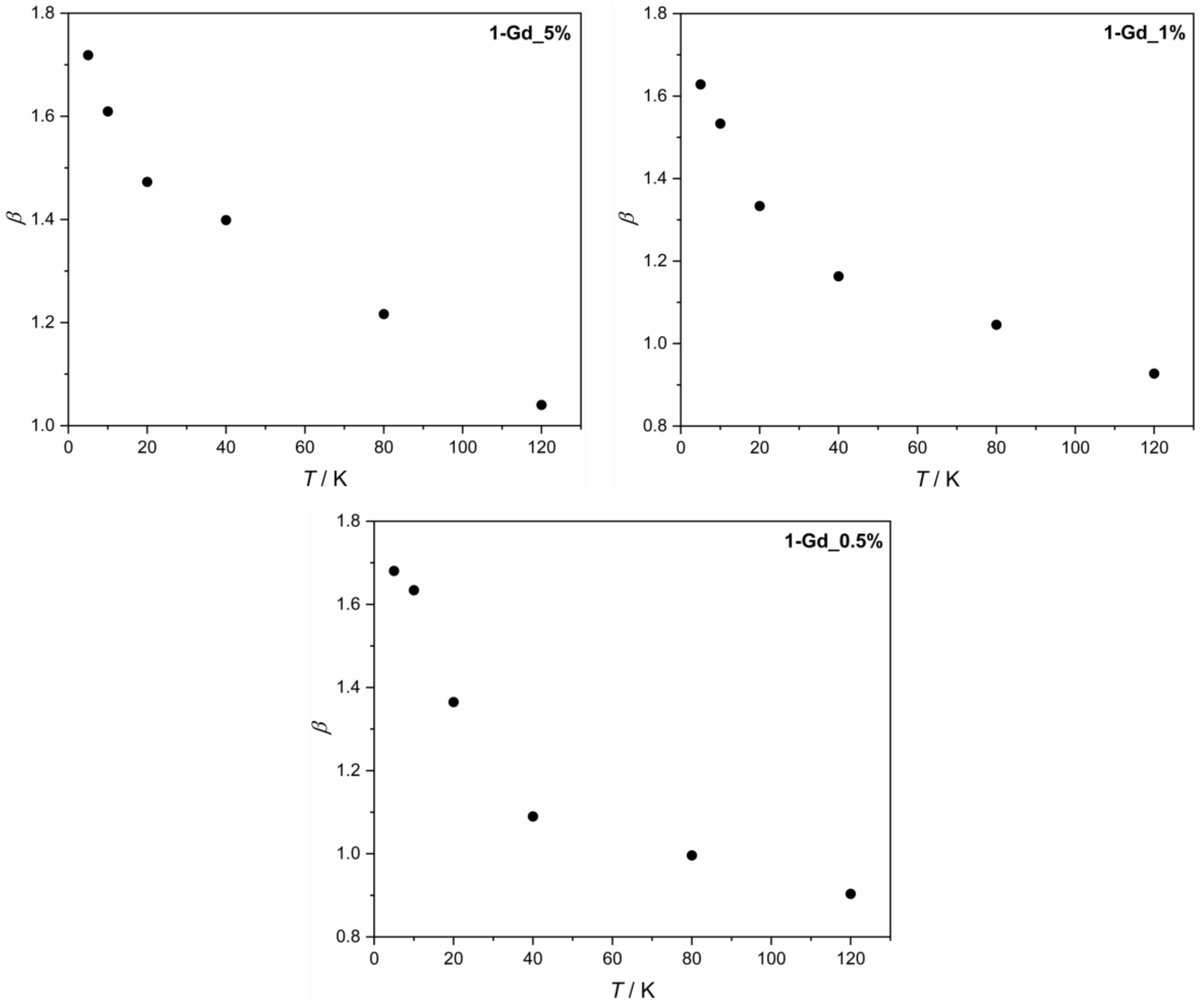

Figure S26. Temperature dependence of the best-fit values of the stretching parameter, *β*, for **1-Gd_5%**, **1-Gd_1%** and **1-Gd_0.5%**.

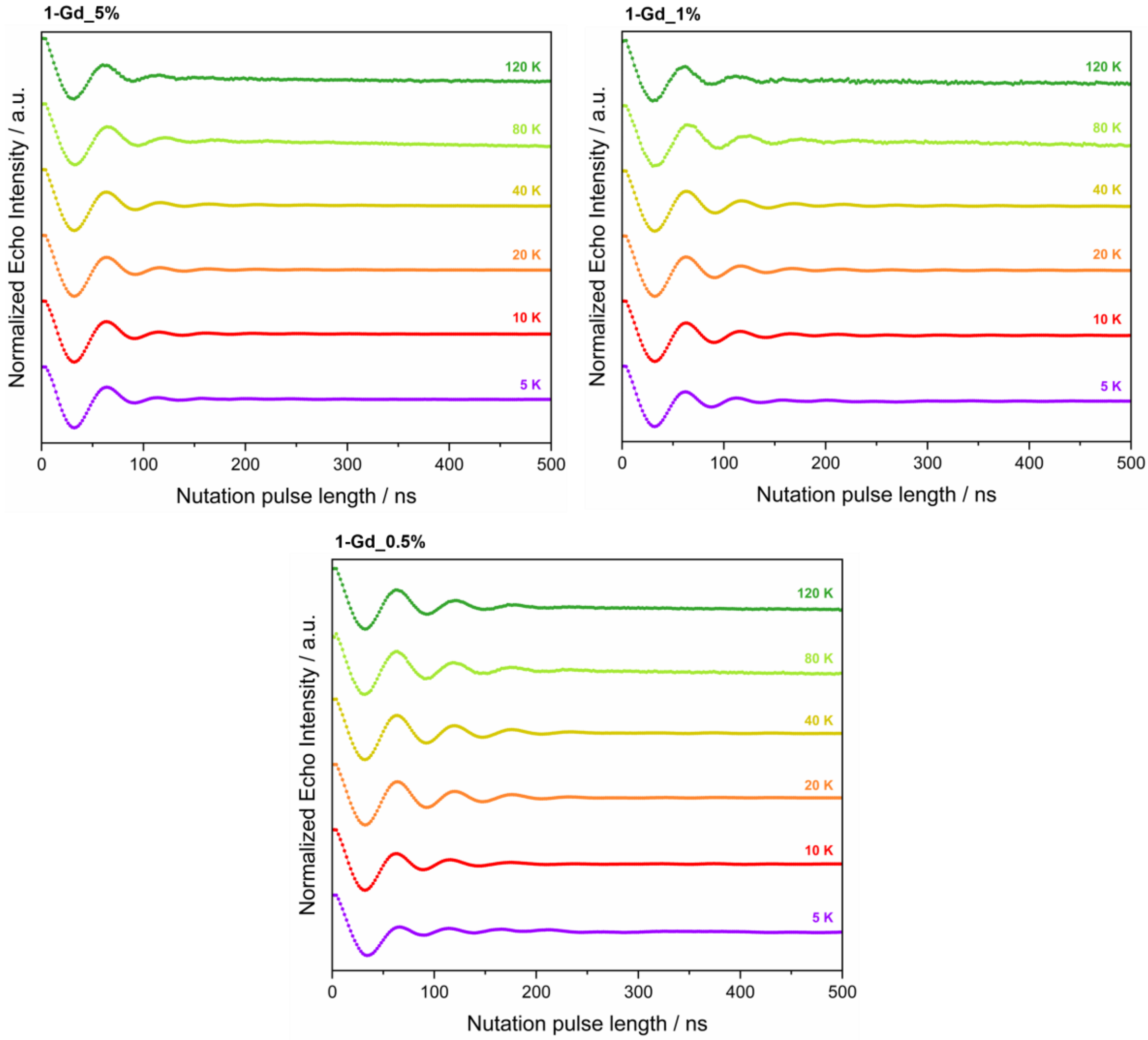

Figure S27. Rabi oscillations recorded at B_0 = 12184 G and different temperatures (microwave attenuation: 14 dB) for the three magnetically diluted samples, **1-Gd_5%**, **1-Gd_1%** and **1-Gd_0.5%**.

Table S14. Comparison of the structural and magnetic properties of the mononuclear Gd^{III}-based complexes reported in the literature showing slow relaxation of the magnetization.

Compound	Closest ideal geometry[a]	H_{dc} / Oe	ν / Hz[b]	T / K[c]	Proposed relaxation mechanism	Ref.
**[Gd(bbpen)Cl]**	PBPY-7	4000	~1000	10	Direct and PB	1
**$[Gd(\alpha\text{-}SiW_{11}O_{39})(H_2L^1)]\cdot 14H_2O$**	BTPR-8 distorted towards SAPR-8	1000	10000	6	Direct and PB	2
**$[Gd(\alpha\text{-}GeW_{11}O_{39})(H_2L^1)]\cdot 14H_2O$**	BTPR-8 distorted towards SAPR-8	2500	10000	8	Direct and PB	3
**$[Gd(pyr)_2(H_2O)_4]Cl_3\cdot 2H_2O$**	TDD-8	2500	10000	6.8	2 Orbach and QTM	4
**$[Gd(phendo)_4](NO_3)_3\cdot xMeOH$**	SAPR-8	3000	1000	3.5	Raman	5
**$[Gd(W_5O_{18})_2]^{9-}$**	SAPR-8	0	~1000	0.1	Orbach and QTM	6
**$[Gd(P_5W_{30}O_{110})]^{12-}$**	JCPAPR-11	0	~100000	0.1	Undetermined	6
**$[GdY(obPC)_3]$**	SAPR-8	2000	1000	10	Direct and PB	7
**$[Gd(THD)_3phen]$**	SAPR-8	8000	1000	5	PB at H_{dc} < 1 T<br>Direct at H_{dc} > 2 T	8
**$[Gd(THD)_3bpy]$**	SAPR-8	8000	1000	10	PB at H_{dc} < 1 T<br>Direct at H_{dc} > 2 T	8
**$Na[Gd(4\text{-}HOpa)_4(H_2O)]\cdot 2H_2O$**	CSAPR-9	1000	10000	10	Direct and Raman	9
**$[Gd(HL^2)_4(DMSO)]\cdot 2H_2O$**	CSAPR-9	1000	10000	4	PB	10
**$[Gd(HL^3)_4(DMSO)]\cdot 3H_2O$**	CSAPR-9	1000	10000	6	PB	10
**$[Gd(18\text{-}c\text{-}6)Cl_4SQ(NO_3)]\cdot I_3$**	JSPC-10	2000	10000	6	Raman and QTM	11
**$[Gd(L^4)(NO_3)_3]\cdot MeOH$**	JSPC-10	4000	1488	5	Spin–phonon relaxation	12

Abbreviations. H_2bbpen = *N,N'*-bis(2-hydroxybenzyl)-*N,N'*-bis(2-picolyl)ethylenediamine; H_2L^1 = *N,N'*-dimethyl-*N,N'*-bis(2-hydroxy-3-formyl-5-bromobenzyl)ethylenediamine; pyr = pyridoxine; phendo = 1,10-phenanthroline-*N,N'*-dioxide; MeOH = methanol; H_2obPC = 2,3,9,10,16,17,23,24-octabutoxyphthalocyanine; THD = 2,2,6,6-tetramethylheptane-3,5-dione; phen = 1,10-phenanthroline; bpy = 2,2'-bipyridyl; 4-HOpa = *N*-4-hydroxyphenyloxamate; H_2L^2 = *N*-(4-Clphenyl)oxamic acid; DMSO = dimethyl sulfoxide; H_2L^3 = *N*-(4-Fphenyl)oxamic acid; 18-c-6 = 1,4,7,10,13,16-hexaoxacyclooctadecane; $Cl_4SQ^{\cdot -}$ = tetrahalo-1,2-semiquinonate; L^4 = N,N'-bis(pyridin-2-ylmethylene)cyclohexane-1,2-diamine. PB = Phonon bottleneck.

[a] The coordination geometry around each Gd^{III} ion has been determined using SHAPE software. *Abbreviations*: PBPY-7: Pentagonal bipyramid (D_{5h}); BTPR-8: Biaugmented trigonal prism (C_{2v}); SAPR-8: Square antiprism (D_{4d}); TDD-8: Triangular dodecahedron (D_{2d}); JCPAPR-11: Capped pentagonal antiprism (C_{5v}); CSAPR-9: Capped square antiprism (C_{4v}); JSPC-10: Sphenocorona (C_{2v}).

[b] Maximum measured frequency value.

[c] Maximum temperature value at which the slow relaxation of the magnetization is observed.

Table S15. Comparison of the structural and magnetic properties of the heterometallic 3d-4f complexes (bearing only one Gd^{III} centre in their composition) reported in the literature showing slow relaxation of the magnetization.

**Compound**	**Closest ideal geometry for Gd^{III}[a]**	**Closest ideal geometry for 3d metal[a]**	**H_{dc} / Oe**	**ν / Hz[b]**	**T / K[c]**	**Proposed relaxation mechanism**	**Ref.**
**$[NiGd(L^1)(NO_3)_3]$**	JSPC-10[d]	SP-4	4000	1488	5	PB	13
**[Ni(*o-van-en*)$GdCl_3(H_2O)$]**	BTPR-8	SP-4	3000	1000	6.5	Undetermined	14
**$[NiGd(H_2L^2)(NO_3)_3]\cdot 4MeOH$**	TD-10	SP-4	5000	1500	6	Direct, Raman and weak QTM	15
**$[Zn(\mu\text{-}L^3)(\mu\text{-}OAc)Gd(NO_3)_2]$**	MFF-9[d]	SPY-5	1000	10000	6	Direct and PB	16
**$[Gd(MgL^4)_2]CF_3SO_3\cdot 0.5H_2O$**	IC-12	OC-6	1000	10000	12	Raman and Direct	17
**$[Gd(ZnL^4)_2]CF_3SO_3\cdot 0.5H_2O$**	IC-12	OC-6	1000	10000	12	Raman and Direct	17
**1-Gd**	SAPR-8	SPY-5	1500	10000	10	Direct and PB	This work

Abbreviations. H_2L^1 = *N*,*N*'-bis(3-methoxysalicylidene)-1,2-diphenylenediamine; H_2(*o-van-en*) = *N*,*N*'-ethylene-bis(3-methoxysalicylaldiminate; H_2L^2 = (3,3′-((1*E*,1′*E*)-(ethane-1,2-diylbis-(azaneylylidene))bis(methaneylylidene))bis(benzene-1,2-diol); H_2L^3 = *N*,*N*'-dimethyl-*N*,*N*'-bis(2-hydroxy-3-methoxy-5-methylbenzyl)-ethylenediamine; H_3L^4 = tris(((2-hydroxy-3-,ethoxybenzyl)-amino)ethyl)amine. PB = Phonon bottleneck.

[a] The coordination geometry around each Gd^{III}/3d ion has been determined using SHAPE software. *Abbreviations*: JSPC-10: Sphenocorona (C_{2v}); BTPR-8: Biaugmented trigonal prism (C_{2v}); TD-10: Tetradecahedron (C_{2v}); MFF-9: Muffin (C_s); IC-12: Icosahedron (I_h); SAPR-8: Square antiprism (D_{4d}); SP-4: Square (D_{4h}); SPY-5: Square pyramid (C_{4v}); OC-6: Octahedron (O_h).

[b] Maximum measured frequency value.

[c] Maximum temperature value at which the slow relaxation of the magnetization is observed.

[d] Crystal structure not reported. The identity of the synthesized species was confirmed by comparison of X-ray crystallographic analysis with that of reported Ln^{III}-based analogues. The coordination geometry has been determined for the latter.

Table S16. Comparison of the structural properties and phase memory times for Gd^{III}-based molecular qubits reported in bibliography.

Compound	Closest ideal geometry[a]	*T* / K	T_m / μs[b]	Dilution method	Ref.
**$[Gd(H_2O)P_5W_{30}O_{110}]^{12-}$**	JCPAPR-11	6	0.47-0.60	Magnetic dilution	18
**$K_5[Gd(\alpha\text{-}SiW_{11}O_{39})(H_2L)]\cdot 14H_2O$**	BTPR-8 towards SAPR-8	3	2.513	Magnetic dilution	19
**$K_5[Gd(\alpha\text{-}GeW_{11}O_{39})(H_2L)]\cdot 14H_2O$**	BTPR-8 towards SAPR-8	3	2.6	Magnetic dilution	19
**$Gd_2@C_{79}N$**	-[c]	5	1.6	Frozen solution	20
**$Gd_2@C_{82}$**	-[d]	5	0.564	Frozen solution	21
**$Gd_2@C_{82}$(morpholine)$_5$**	-[d]	5	5.1	Frozen solution	21
**$Gd_2@C_{82}$(morpholine)$_7$**	-[d]	5	4.4	Frozen solution	21
**$Gd_2@C_{82}$(morpholine)$_9$**	-[d]	5	4.5	Frozen solution	21
**(Hpy)$[Gd_2(HL)_3(NO_3)(py)(H_2O)]\cdot 5py$**	MFF-9 / CSAPR-9[e]	6	0.73	Frozen solution	22
**(Hpy)$[LaGd(HL)_3(NO_3)(py)(H_2O)]\cdot 5py$**	TCTPR-9 towards CSAPR-9	6	1.05	Frozen solution	22
**(Hpy)$[GdLu(HL)_3(NO_3)(py)(H_2O)]\cdot 5py$**	MFF-9	6	1.31	Frozen solution	22
**[Gd(phendo)$_4$]$(NO_3)_3\cdot$xMeOH**	SAPR-8	5	2.7	Magnetic dilution	5
**$[NEt_4][Gd\{Pt(SAc)_4\}_2]$**	SAPR-8	10	1.14	Magnetic dilution	23
**[Gd(bipyNO)$_4$](TfO)$_3\cdot$xMeOH**	SAPR-8	3.5	0.612	Magnetic dilution	24
***s*-P2-Gd**	CTPR-7 towards COC-7	3	~8	Frozen solution	25
***f*-P2-Z·Gd_2**	-[f]	3	9.8	Frozen solution	25
***f*-P2-E·Gd_2**	-[f]	3	8.2	Frozen solution	25
**Gd(trensal)**	COC-7	3	12	Magnetic dilution	26
**Gd(trenovan)**	COC-7[g]	5	4.5	Magnetic dilution	27
**Gd(trenpvan)**	COC-7[g]	5	1.9	Magnetic dilution	27
**Na[Gd(4-HOpa)$_4$(H_2O)]$\cdot 2H_2O$**	CSAPR-9	8	4.25	Magnetic dilution	28
**$[N(C_2H_5)_4][Gd(CAN)_2]$**	SAPR-8	3.2	1.957	Magnetic dilution	29
**Gd-DOTA**	CSAPR-9	14	3.74	Frozen solution	30
**Iodo-(Gd-PyMTA)**	-[f]	12	3.59	Frozen solution	30
**[(por^1)Gd(Klaui)]**	CTPR-7	3	3.6	Frozen solution	31
**[(por^2)$_2$Gd$_2$(Klaui)$_2$]**	COC-7	3	3	Frozen solution	31

Table S16 (continuation). Comparison of the structural properties and phase memory times for Gd^{III}-based molecular qubits reported in bibliography.

Compound	Closest ideal geometry[a]	*T* / K	T_m / μs[b]	Dilution method	Ref.
**$[(Gd)_3(m\text{CB-L})_4(NO_3)(DMF)_x]_n$**	BTPR-8 / CTPR-7 / SAPR-8[e, g]	3.6	0.716	Magnetic dilution	32
**$[Gd_2(pydc)_6(H_2pip)_3]\cdot 20H_2O$**	TCTPR-9[g]	5	0.994	Magnetic dilution	33
**1-Gd**	SAPR-8	5	4.938	Magnetic dilution	This work

Abbreviations. H_2L = *N*,*N*'-dimethyl-*N*,*N*'-bis(2-hydroxy-3-formyl-5-bromobenzyl)ethylenediamine; H_3L = 6-(3-oxo-3-(2-hydroxyphenyl)propionyl)-pyridine-2-carboxylic acid; py = pyridine; phendo = 1,10-phenanthroline-*N*,*N*'-dioxide; SAc = thioacetate; bipyNO = 4,4-bipyridine-*N*,*N*'-dioxide; TfO = triflate; H_3trensal = 2,2',2"-tris(salicylideneimino)triethylamine; H_3trenovan = tris(((3-methoxysalicylidene)amino)ethyl)amine; H_3trenpvan = tris(((5-methoxysalicylidene)amino)ethyl)amine; 4-HOpa = *N*-4-hydroxyphenyloxamate; CAN^{2-} = chloranilate; DOTA = 1,3,7,10-tetraazacyclododecane-1,4,7,10-tetraacetic acid; PyMTA = 2,2',2'',2'''-[(pyridine-2,6-diyl)bis(methylenenitrilo)]tetrakis(acetic acid); por[1] = 5,15-bis(3,5-di-t-butylphenyl)-10,20-bis{[tri-isopropylsilyl]ethynyl}porphyrin; $Klaui^-$ = tris(μ-diethyl posphonato)-(η^5-cyclopentadienyl)-cobaltato; por[2] = tris(μ-diethyl phosphonato)-(η^5-cyclopentadienyl)-(5,15-bis(3,5-di-t-butylphenyl)-10-(tri-isopropylsilylethynyl)porphyrin-20-yl; *m*CB-L = 1,7-di(4-carboxyphenyl)-1,7-dicarba-*closo*-dodecarborane; DMF = *N*,*N*-dimethylformamide: pydc = pyridine-2,6-dicarboxylic acid; H_2pip = piperazine.

[a] The coordination geometry around each Gd^{III} ion has been determined using SHAPE software. *Abbreviations*: JCPAPR-11: Capped pentagonal antiprism (C_{5v}); BTPR-8: Biaugmented trigonal prism (C_{2v}); SAPR-8: Square antiprism (D_{4d}); MFF-9: Muffin (C_s); CSAPR-9: Capped square antiprism (C_{4v}); TCTPR-9: Tricapped trigonal prism (D_{3h}); MFF-9: Muffin (C_s); CTPR-7: Capped trigonal prism (C_{2v}); COC-7: Capped octahedron (C_{3v}).

[b] Highest reported T_m value.

[c] Gd^{III} ions are encapsulated in an azafullerene.

[d] Gd^{III} ions are encapsulated in an endohedral fullerene.

[e] Several crystallographically independent Gd^{III} ions in the unit cell.

[f] Crystal structure not obtained or not uploaded to the CCDC.

[g] Crystal structure not reported. The identity of the synthesized species was confirmed by comparison of X-ray crystallographic analysis with that of reported Ln^{III}-based analogues. The coordination geometry has been determined for the latter.

References

(1) Chen, Y. C.; Peng, Y. Y.; Liu, J. L.; Tong, M. L. Field-Induced Slow Magnetic Relaxation in a Mononuclear Gd(III) Complex. *Inorg. Chem. Commun.* **2019**, *107*, 107449. DOI:10.1016/j.inoche.2019.107449.

(2) Ruiz-Bilbao, E.; Pardo-Almanza, M.; Oyarzabal, I.; Artetxe, B.; Felices, L. S.; García, J. A.; Seco, J. M.; Colacio, E.; Lezama, L.; Gutiérrez-Zorrilla, J. M. Slow Magnetic Relaxation and Luminescent Properties of Mononuclear Lanthanide-Substituted Keggin-Type Polyoxotungstates with Compartmental Organic Ligands. *Inorg. Chem.* **2022**, *61*, 2428–2443. DOI:10.1021/acs.inorgchem.1c03214.

(3) Bustamante-Fernández, J.; Ruiz-Bilbao, E.; Rodríguez-Esteban, C.; Gonidec, M.; García, J. A.; Lezama, L.; Gutiérrez-Zorrilla, J. M.; Oyarzabal, I.; Artetxe, B. Effect of the Heteroatom on the Magnetic and Luminescence Properties of Hybrid Lanthanide-Substituted Keggin-Type Polyoxometalates. *Dalton Trans.* **2024**, *53*, 13330–13334. DOI:10.1039/d4dt01379d.

(4) Orts-Arroyo, M.; Ten-Esteve, A.; Ginés-Cárdenas, S.; Cerdá-Alberich, L.; Martí-Bonmatí, L.; Martínez-Lillo, J. A Gadolinium(III) Complex Based on Pyridoxine Molecule with Single-Ion Magnet and Magnetic Resonance Imaging Properties. *Int. J. Mol. Sci.* **2024**, *25*, 2112. DOI:10.3390/ijms25042112.

(5) Handzlik, G.; Magott, M.; Arczyński, M.; Sheveleva, A. M.; Tuna, F.; Sarewicz, M.; Osyczka, A.; Rams, M.; Vieru, V.; Chibotaru, L. F.; Pinkowicz, D. Magnetization Dynamics and Coherent Spin Manipulation of a Propeller Gd(III) Complex with the Smallest Helicene Ligand. *J. Phys. Chem. Lett.* **2020**, *11*, 1508–1515. DOI:10.1021/acs.jpclett.9b03275.

(6) Martínez-Pérez, M. J.; Cardona-Serra, S.; Schlegel, C.; Moro, F.; Alonso, P. J.; Prima-García, H.; Clemente-Juan, J. M.; Evangelisti, M.; Gaita-Ariño, A.; Sesé, J.; Van Slageren, J.; Coronado, E.; Luis, F. Gd-Based Single-Ion Magnets with Tunable Magnetic Anisotropy: Molecular Design of Spin Qubits. *Phys. Rev. Lett.* **2012**, *108*, 247213. DOI:10.1103/PhysRevLett.108.247213.

(7) Horii, Y.; Katoh, K.; Miyazaki, Y.; Damjanović, M.; Sato, T.; Ungur, L.; Chibotaru, L. F.; Breedlove, B. K.; Nakano, M.; Wernsdorfer, W.; Yamashita, M. Coexistence of Spin–Lattice Relaxation and Phonon-Bottleneck Processes in Gd^{III}–Phthalocyaninato Triple-Decker Complexes under Highly Diluted Conditions. *Chem. Eur. J.* **2020**, *26*, 8076–8082. DOI:10.1002/chem.201905796.

(8) Qian, K.; Wang, B.; Wang, Z.; Su, G.; Gao, S. Field-Dependent Magnetic Relaxation and Magnetocaloric Effect in Mononuclear Gd Complexes. *Acta Chim. Sin.* **2013**, *71*, 1022–1028. DOI:10.6023/A13030319.

(9) da Cunha, T. T.; Barbosa, V. M. M.; Oliveira, W. X. C.; Pinheiro, C. B.; Pedroso, E. F.; Nunes, W. C.; Pereira, C. L. M. Slow Magnetic Relaxation in Mononuclear Gadolinium(III) and Dysprosium(III) Oxamato Complexes. *Polyhedron* **2019**, *169*, 102–113. DOI:10.1016/j.poly.2019.04.056.

(10) Vaz, R. C. A.; Esteves, I. O.; Oliveira, W. X. C.; Honorato, J.; Martins, F. T.; Marques, L. F.; Dos Santos, G. L.; Freire, R. O.; Jesus, L. T.; Pedroso, E. F.; Nunes, W. C.; Julve, M.; Pereira, C. L. M. Mononuclear Lanthanide(III)-Oxamate Complexes as New Photoluminescent Field-Induced Single-Molecule Magnets: Solid-State Photophysical and Magnetic Properties. *Dalton Trans.* **2020**, *49*, 16106–16124. DOI:10.1039/d0dt02497j.

(11) Dunstan, M. A.; Brown, D. S.; Sorace, L.; Mole, R. A.; Boskovic, C. Modulation of Slow Magnetic Relaxation in Gd(III)-Tetrahalosemiquinonate Complexes. *Chem. Asian J.* **2022**, *17*, e202200325. DOI:10.1002/asia.202200325.

(12) Pilichos, E.; Font-Bardia, M.; Escuer, A.; Mayans, J. Structural and magnetic studies of mononuclear lanthanide complexes derived from N-rich chiral Schiff bases. *Dalton Trans.*, **2021**, *50*, 1746–1753. DOI: 10.1039/d0dt04224b.

(13) Mayans, J.; Escuer, A. Correlating the Axial Zero Field Splitting with the Slow Magnetic Relaxation in Gd^{III} SIMs. *Chem. Commun.* **2021**, *57*, 721–724. DOI:10.1039/d0cc07474h.

(14) Vráblová, A.; Tomás, M.; Falvello, L. R.; Dlháň, Ľ.; Titiš, J.; Černák, J.; Boča, R. Slow Magnetic Relaxation in Ni-Ln (Ln = Ce, Gd, Dy) Dinuclear Complexes. *Dalton Trans.* **2019**, *48*, 13943–13952. DOI:10.1039/c9dt02122a.

(15) Costa-Villén, E.; Font-Bardia, M.; Mayans, J.; Escuer, A. Field-Induced Slow Relaxation of the Magnetization in Two Families of [$M^{II}Ln^{III}$] Complexes. *Cryst. Growth Des*. **2024**, *24*, 5806−5817. DOI: 10.1021/acs.cgd.4c00598.

(16) Zabala-Lekuona, A.; Cepeda, J.; Oyarzabal, I.; Rodríguez-Diéguez, A.; García, J. A.; Seco, J. M.; Colacio, E. Rational Design of Triple-Bridged Dinuclear $Zn^{II}Ln^{III}$-Based Complexes: A Structural, Magnetic and Luminescence Study. *CrystEngComm* **2017**, *19*, 256–264. DOI:10.1039/c6ce02240e.

(17) Masuda, Y.; Sakata, S.; Kayahara, S.; Irie, N.; Kofu, M.; Kono, Y.; Sakakibara, T.; Horii, Y.; Kajiwara, T. Slow Magnetic Relaxation of Linear Trinuclear M(II)-Gd(III)-M(II) Complexes with D_3 Point Group Symmetry (M(II) = Zn(II) and Mg(II)). *J. Phys. Chem. C* **2023**, *127*, 3295–3306. DOI:10.1021/acs.jpcc.2c08323.

(18) Jenkins, M. D.; Duan, Y.; Diosdado, B.; García-Ripoll, J. J.; Gaita-Ariño, A.; Giménez-Saiz, C.; Alonso, P. J.; Coronado, E.; Luis, F. Coherent Manipulation of Three-Qubit States in a Molecular Single-Ion Magnet. *Phys. Rev. B* **2017**, *95*, 064423. DOI:10.1103/PhysRevB.95.064423.

(19) Rodríguez-Esteban, C.; Ruiz-Bilbao, E.; Bustamante-Fernández, J.; Tuna, F.; Lezama, L.; Mailman, A.; Ullah, A.; Gaita-Ariño, A.; Artetxe, B.; Gonidec, M.; Oyarzabal, I. Coherent Spin Manipulation in Mononuclear Gadolinium-Substituted Polyoxometalate–Organic Hybrids. *J. Phys. Chem. Lett.* **2025**, *16*, 3072–3077. DOI:10.1021/acs.jpclett.5c00196.

(20) Hu, Z.; Dong, B. W.; Liu, Z.; Liu, J. J.; Su, J.; Yu, C.; Xiong, J.; Shi, D. E.; Wang, Y.; Wang, B. W.; Ardavan, A.; Shi, Z.; Jiang, S. D.; Gao, S. Endohedral Metallofullerene as Molecular High Spin Qubit: Diverse Rabi Cycles in $Gd_2@C_{79}N$. *J. Am. Chem. Soc.* **2018**, *140*, 1123–1130. DOI:10.1021/jacs.7b12170.

(21) Liu, Z.; Huang, H.; Wang, Y. X.; Dong, B. W.; Sun, B. Y.; Jiang, S. D.; Gao, S. Amination of the $Gd@C_{82}$ Endohedral Fullerene: Tunable Substitution Effect on Quantum Coherence Behaviors. *Chem. Sci.* **2020**, *11*, 10737–10743. DOI:10.1039/d0sc02182b.

(22) Luis, F.; Alonso, P. J.; Roubeau, O.; Velasco, V.; Zueco, D.; Aguilà, D.; Martínez, J. I.; Barrios, L. A.; Aromí, G. A Dissymmetric [Gd_2] Coordination Molecular Dimer Hosting Six Addressable Spin Qubits. *Commun. Chem.* **2020**, *3*, 176. DOI:10.1038/s42004-020-00422-w.

(23) Fang, Y.-H.; Liu, Z.; Wang, Y.-X.; Zhou, S.; Jiang, S.-D.; Gao, S. Orientation Mapping of Rabi Frequencies in a Rare-Earth Molecular Qudit. *Inorg. Chem. Front.* **2020**, *7*, 3875–3881. DOI:10.1039/d0qi00784f.

(24) López-Cabrelles, J.; Escalera-Moreno, L.; Hu, Z.; Prima-García, H.; Espallargas, G. M.; Gaita-Ariño, A.; Coronado, E. Near Isotropic D_{4d} Spin Qubits as Nodes of a Gd(III)-Based Metal-Organic Framework. *Inorg. Chem.* **2021**, *60*, 8575–8580. DOI:10.1021/acs.inorgchem.1c00504.

(25) Van Raden, J. M.; Alexandropoulos, D. I.; Slota, M.; Sopp, S.; Matsuno, T.; Thompson, A. L.; Isobe, H.; Anderson, H. L.; Bogani, L. Singly and Triply Linked Magnetic Porphyrin Lanthanide Arrays. *J. Am. Chem. Soc.* **2022**, *144*, 8693–8706. DOI:10.1021/JACS.2C02084.

(26) Buch, C. D.; Kundu, K.; Marbey, J. J.; Van Tol, J.; Weihe, H.; Hill, S.; Piligkos, S. Spin-Lattice Relaxation Decoherence Suppression in Vanishing Orbital Angular Momentum Qubits. *J. Am. Chem. Soc.* **2022**, *144*, 17597–17603. DOI:10.1021/jacs.2c07057.

(27) Hansen, S. H.; Buch, C. D.; Weihe, H.; Piligkos, S. Effect of Chemical Modification in Trigonal Gd(III) Molecular Qubits. *Inorg. Chem.* **2025**, *64*, 4912–4919. DOI:10.1021/acs.inorgchem.4c04789.

(28) Wang, J.; Jing, Y.; Cui, M.-H.; Lu, Y.-M.; Ouyang, Z.; Shao, C.; Wang, Z.; Song, Y. Spin Qubit in a 2D $Gd^{III}Na^{I}$-Based Oxamato Supramolecular Coordination Framework. *Chem. Eur. J.* **2023**, *29*, e202301771. DOI:10.1002/chem.202301771.

(29) Du, X.; Sun, L. Optimizing the Spin Qubit Performance of Lanthanide-Based Metal-Organic Frameworks. *Inorg. Chem. Front.* **2024**, *11*, 8660–8670. DOI:10.1039/d4qi02324b.

(30) Wilson, C. B.; Qi, M.; Han, S.; Sherwin, M. S. Gadolinium Spin Decoherence Mechanisms at High Magnetic Fields. *J. Phys. Chem. Lett.* **2023**, *14*, 10578–10584. DOI:10.1021/acs.jpclett.3c01847.

(31) Le Roy, J. J.; Cremers, J.; Thomlinson, I. A.; Slota, M.; Myers, W. K.; Horton, P. H.; Coles, S. J.; Anderson, H. L.; Bogani, L. Tailored Homo- and Hetero-Lanthanide Porphyrin Dimers: A Synthetic Strategy for Integrating Multiple Spintronic Functionalities Into a Single Molecule. *Chem. Sci.* **2018**, *9*, 8474–8481. DOI:10.1039/c8sc03762k.

(32) Bartolomé, E.; Li, X. B.; Arauzo, A.; Luzón, J.; García-Rubio, I.; Planas, J. G. A Triple-Site Gd_3 Carborane Metal-Organic Framework toward Scalable Quantum Computing. *ACS Appl. Mater. Interfaces* **2025**, *17*, 42082–42095. DOI:10.1021/acsami.5c06002.

(33) Xu, X.-N.; Zhang, Y.-S.; Song, J.-M.; Deng, Q.-S.; Liao, Y.-Q.; Yuan, J.-Y.; Gao, S.; Zhou, S.; Jiang, S.-D. A High-Dimensional Spin-Qudit Candidate of Gd(III) Center Complex Crystal with Addressable Transitions. *CCS Chem.* **2024**, *7*, 2372–2380. DOI:10.31635/ccschem.024.202404786.
